# Environmental Profile of a Community's Health (EPOCH): An Instrument to Measure Environmental Determinants of Cardiovascular Health in Five Countries

**DOI:** 10.1371/journal.pone.0014294

**Published:** 2010-12-10

**Authors:** Clara K. Chow, Karen Lock, Manisha Madhavan, Daniel J. Corsi, Anna B. Gilmore, S. V. Subramanian, Wei Li, Sumathi Swaminathan, Patricio Lopez-Jaramillo, Alvaro Avezum, Scott A. Lear, Gilles Dagenais, Koon Teo, Martin McKee, Salim Yusuf

**Affiliations:** 1 Population Health Research Institute, Hamilton Health Sciences & McMaster University, Hamilton, Canada; 2 The George Institute for Global Health and Sydney Medical School, University of Sydney, Sydney, Australia; 3 European Centre on Health of Societies in Transition, London School of Hygiene and Tropical Medicine, London, United Kingdom; 4 Department of Society, Human Development and Health, Harvard School of Public Health, Boston, Massachusetts, United States of America; 5 Cardiovascular Institute & Fuwai Hospital, Chinese Academy of Medical Sciences, Beijing, China; 6 St. John's Research Institute, Bangalore, Karnataka, India; 7 Research Direction, Fundacion Oftalmologica de Santander-Clinica Carlos Arila Lulle, and Medical School, Universidad de Santander, Bucaramanga, Colombia; 8 Dante Pazzanese Institute of Cardiology, São Paulo, Brazil; 9 Faculty of Health Sciences, Simon Fraser University, Vancouver, Canada; 10 Department for Health, University of Bath, Bath, United Kingdom; 11 Institut Universitaire de Cardiologie et de Pneumologie de Québec, Université Laval, Quebec City, Canada; Yale University School of Medicine, United States of America

## Abstract

**Background:**

The environment in which people live is known to be important in influencing diet, physical activity, smoking, psychosocial and other risk factors for cardiovascular (CV) disease. However no instrument exists that evaluates communities for these multiple environmental factors and is suitable for use across different communities, regions and countries. This report describes the design and reliability of an instrument to measure environmental determinants of CV risk factors.

**Method/Principal Findings:**

The Environmental Profile of Community Health (EPOCH) instrument comprises two parts: (I) an assessment of the physical environment, and (II) an interviewer-administered questionnaire to collect residents' perceptions of their community. We examined the inter-rater reliability amongst 3 observers from each region of the direct observation component of the instrument (EPOCH I) in 93 rural and urban communities in 5 countries (Canada, Colombia, Brazil, China and India). Data collection using the EPOCH instrument was feasible in all communities. Reliability of the instrument was excellent (Intraclass Correlation Coefficient - ICC>0.75) for 24 of 38 items and fair to good (ICC 0.4–0.75) for 14 of 38 items.

**Conclusion:**

This report shows data collection with the EPOCH instrument is feasible and direct observation of community measures reliable. The EPOCH instrument will enable further research on environmental determinants of health for population studies from a broad range of settings.

## Introduction

It is now generally accepted that the physical and social environment play an important role in influencing the diet, physical activity, smoking, and other health-related behaviours of adults and children.[1] These behavioural risk factors impact directly and indirectly, through intermediate conditions such as obesity, hypertension, abnormal lipid profiles and dysglycaemia, on a range of chronic diseases.

Many instruments have been developed to measure environmental factors that influence health-related behaviours, [2], [3], [4], [5], [6], [7], [8] however most of these have focused on a single health behaviour such as smoking, physical activity or diet and on one aspect of the environment. For example, Joossen's Tobacco Control Scale assesses the presence of policies to reduce smoking but does not include other environmental measures such as social acceptability.[7], [8] Instruments designed to measure various aspects of the ‘food environment’[5] have looked at food stores,[9] restaurants,[10] schools[11] or worksites.[12] Instruments measuring the physical activity environment are the best developed. These can be divided into objective (information obtained through systematic observation, audit or archival geographic data) [3], [13] and subjective (obtained through questioning individuals on the perceptions of their environment).[2], [4] A recent review of physical activity environment instruments identified 20 objective assessment tools and 19 perception-based questionnaires.[6] Measures included in both types of instrument include: the availability of walking infrastructure, neighbourhood crime and safety and presence of local government support e.g. funded parks but few research teams combine perception and objective measures.

Most of the instruments described in the literature have been developed and used in discrete geographic settings, typically in the United States,[14] Australia[15] or the United Kingdom[16] (exceptions are some instruments assessing tobacco policies)[7], [17] and many are likely to be unsuitable or require significant cultural adaptation to be applied elsewhere and particularly in low or middle income countries. Few instruments have been used in rural areas. This is important given the sizeable rural populations in many countries in the world. In addition few have been subject to reliability testing and only one instrument that we identified has been tested for reliability in multiple countries.[18]

To enable a comparative examination of environmental factors and analysis of a broad range of conditions in which environmental factors are likely to be causal such as obesity, diabetes and cardiovascular disease, it will be necessary to develop instruments that measure multiple aspects of the environment in simple and reliable ways. We have been assessing methods that can measure environmental determinants of health in communities from diverse cultural, socioeconomic, and regional (urban and rural) settings of 17 countries as part of the Prospective Urban Rural Epidemiology (PURE) study. [19] As we note above, existing instruments have not been utilised in this range of settings. Within the limited resources of a large epidemiological study our aim was to create an instrument that could collect reliable and comparable data on environmental characteristics associated with cardiovascular risk factors across these diverse communities. This paper describes the design and testing of reliability of the EPOCH (Environmental Profile of a Community's Health) instrument.

## Methods

### EPOCH Instrument development

The EPOCH instrument was developed initially from a review of existing instruments and community level measures that influence cardiovascular risk factors (CVRFs) and this review has been described separately.[1] Four major domains were identified: the tobacco environment, the physical activity environment, the food (including alcohol) environment, and the social and economic environment. Within each domain a list of items that should be included in each was made. Thus items included in the tobacco environment domain were: price of cigarettes, smoke-free zones, tobacco advertising, support available for quitting, health warnings and other information on the harms of tobacco, access to tobacco generally and by youth and social acceptability of smoking. Items included in the physical activity domain were: availability and access to public transport, sidewalks, street lighting, safety of roads, aesthetics of community, availability of and access to local services including recreation facilities and parks, advertising for physical activity and policies and media promoting physical activity. Items included in the food environment were price of high and low nutrition foods, food advertisements, access to and availability of fruit and vegetables, policies and media promoting healthy diets, food labelling. Some measures from the social and economic domains overlapped with other domains; additional measures collected here were other household expenditure such as housing, as well as measures of social support and social cohesion. As far as possible the measures included in the EPOCH instruments sought to be comprehensive within the constraints of an instrument that was practical and feasible to administer. After the pilot phase a few measures were dropped (e.g. the quality of parks, the extent of physical disorder) as these measures were difficult to assess objectively and concerns were raised by many of our in-country investigators that qualitative measures (e.g. Likert scales) would be difficult to compare across communities from different countries.

As the main aim was to create an instrument that was applicable to diverse cultural, socio-economic and regional (urban/rural) settings, researchers from a wide range of the PURE study countries were involved in an iterative process of instrument development. The measures and underlying concepts of the proposed instrument were discussed in a series of face-to-face meetings with investigators and data collectors from each country. From these meetings standard definitions and data collection methods were developed to ensure that our instrument captured the same concepts in each community. In some cases this involved identifying equivalents in different settings. For example stores that sell cigarettes may be stand-alone market stalls in some countries or parts of convenience stores or supermarkets in others. In some cases data collection was limited to basic items to ensure broad applicability. For example, a universal grocery list of common food items was created by identifying common foods from Food Frequency Questionnaires (FFQ) data collected for the main PURE study [20] and this was cross checked against lists of frequently consumed foods available by country from the Food and Agriculture Organisation (http://faostat.fao.org/).

### Methods of data collection used in EPOCH

The instrument was developed in two parts: EPOCH 1 is an objective environmental audit tool in which trained researchers directly observe and systematically record physical aspects of the environment using a pro-forma, with standardized operational definitions. EPOCH 2 is an interviewer administered questionnaire that captures perceptions about the community from PURE subjects living in that community.

**EPOCH 1** has five sections. The first, ‘*Community characteristics*’, is a checklist of essential infrastructure and services in the community. The second, a ‘*Community observation walk*’, takes place in a commercial or central shopping district that people use for everyday purchases. Its precise location is selected on the basis of local knowledge by study coordinators. Researchers walk according to a planned route covering 1 kilometre, beginning from a pre-specified central location designated as the ‘start point’ (e.g. a central busy traffic intersection, central train or bus station, post office, supermarket, shopping mall, school or other central area where people frequently visit). On the walk researchers count the different types of advertisements, shops and note other features of the community environment including the presence and quality of the sidewalk. The walk generally took about 1 hour.

The third section is ‘*Assessment of a tobacco retail outlet*’ and the fourth is an ‘*Assessment of a grocery store*’. The aim of these assessments was to capture price, access to and availability of products, and presence of in-store advertising. The fifth section is an ‘*Assessment of a local restaurant*’. The closest tobacco store, grocery store and local restaurant to the ‘start point’ of the community observation walk were selected for the detailed assessments. If none existed, these were not done.

**EPOCH 2** includes questions that aim to capture, (i) what participants observe in their community; (ii) their awareness of local laws, regulations, and health programs, and (iii) their opinions about behaviours and laws. For example, participants are asked where they have observed individuals smoking in their community or what types of advertisements (for and against smoking) participants have seen in different types of media. Questions are included on whether, in their communities, smoking is currently allowed, and their opinion of social acceptability of smoking.

The feasibility of using the EPOCH instruments was tested initially in 25 rural and urban communities in Brazil, Canada, China, Colombia and India. Quantitative and qualitative information from pre-testing was reviewed by three working groups involving international collaborators which led to further refinements of the instrument. Pre-testing also established the feasibility of data collection by research assistants with only 2 hours of training.

### Data collection

To evaluate the performance of the EPOCH instruments, a convenience sample of 93 other urban and rural communities involved in the PURE study from China (Yunnan, Qinghai, Beijing, Jiangsu, Shandong, Shanxi, Shannxi, Jiangxi, Liaoning, Xinjiang, Sichuan provinces), India (Karnataka state), Colombia (Santander, Nariño, Quindio, Bolivar), Brazil (Sao Paulo, Angatuba, North region) and Canada (British Colombia, Ontario and Quebec) were selected. Communities in the PURE study were selected by local country investigators to align with administrative borders (such as census tracts or postal zones). For example in Canada community boundaries was based on an area (suburb or town) name and the corresponding cluster of postal codes. In rural areas in India, China or Colombia it was village boundaries. In urban areas, selected urban communities in each country were sampled across different local income strata to capture within country diversity (Table 1).[19]

**Table 1:** Characteristics of communities surveyed.

Characteristics	Brazil	Canada	China	Colombia	India
Number of communities	6	39	19	13	15
Rural (%)	50	33.3	26.3	42.9	66.7
Paved roads (%)	66.7	100	100	92.9	93.3
Traffic lights (%)	50	97	68.4	42.9	26.7
Highway in community allowing speeds >50 km/hr (%)	66.7	84.6	26.3	7.1	20.0
Availability of Internet access	50	100	94.4	100	26.7

Manuals and training slides were translated and distributed prior to a two hour training session. Face-to-face training was conducted in China, India and the Ontario site in Canada and training at these sites involved a session where all observers and the trainer visited at least one community together to do an assessment. Teleconference and web conferencing were used for other sites and in these sites community observers made at least one practice assessment prior to commencing the study. Three researchers from each recruiting site were trained to administer EPOCH I. Each assessment was undertaken independently at a similar time of day and within two weeks of the first assessment between May 2008 and March 2009. At the end of the study researchers were asked to give qualitative feedback on the conduct and feasibility of data collection. EPOCH 1 and 2 instruments and manual of operations are available in Appendix S1, S2, S3. All training was conducted by the lead author.

#### Ethics statement

The EPOCH instruments were approved by the Hamilton Health Sciences/McMaster Health Sciences Research Ethics board. Written informed consent was obtained from all participants in the study.

### Analysis

The inter-rater reliability of the objective component of the EPOCH tool across all communities and in major sub-groups was assessed. The EPOCH-1 reliability study was conducted in which a sample of *k* observers measured *n* community-level characteristics from 93 communities in the EPOCH pilot countries (Canada, India, China, Colombia, and Brazil). The *j*th independent assessment of the *i*th community-level characteristic, X*_ij_*, is represented under the two-way random effects model as:
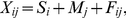
(1)


Where S*_i_* is the effect of the community-level characteristic (assumed to be normally distributed with mean 0 and variance 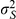); M*_j_* is the random effect of assessment *j*, and F*_ij_* is the random error associated with this particular community-level characteristic (assumed to be normally distributed with mean 0 and variance 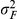). Under this model, it is assumed that all variables are mutually independent and that there is no observer-by-community characteristic interaction [21]. The intraclass correlation coefficient (ICC) is given by: 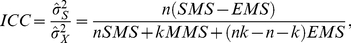
(2)where SMS, MMS, and EMS are the mean squares for community-level characteristics, assessments, and error respectively, obtained from the two way analysis of variance (ANOVA) design [22].

We classified ICC above 0.75 as excellent agreement and below 0.4 as poor agreement.[23] Reliability is reported for the entire group and was also calculated for sub-groups (urban communities versus rural, by country, and by country economic level).

Analyses were conducted using STATA version 11.0.

## Results

### Feasibility

In general, observers reported few problems. For EPOCH 1, they reported that the majority of items were “easy” to collect by observers and that assessments became easier with experience. In-person training and conduct of test community assessments with the trainer was helpful to discuss definitions and explain concepts, particularly in areas where English was not widely spoken. Observers in India asked about large seasonal variations in fruit and vegetable availability, while those in some communities from Canada raised questions regarding the price of housing. In Canada, secondary data sources usually only reported the average cost of residential housing for a larger district that may not correspond to the smaller community being evaluated. This variable was hence left missing for a number of Canadian communities. With EPOCH 2, interviewers noted differences in the understanding of the term ‘community’. Interviewers identified the need to include an introductory paragraph setting out how the “community” was defined.

### EPOCH 1 items

The frequency of certain observations was consistent with prior expectations, giving face validity. For example the communities in China recorded the most tobacco advertisements and communities in Canada the least; communities in Canada and Colombia recorded the most snack food advertisements and communities in China and India the least. Communities from China also recorded the most outlets that sold cigarettes and communities from Canada the least. Communities in urban Canada reported the highest number of infrastructure and health facilities and communities from rural India and China the least. Communities in urban Canada also reported the greatest range of fruits and vegetables and communities in rural India and China the least. Incompleteness of items was often due to items being not available in communities. In India, there were no restaurants in 5 communities. A number of items could not be priced as they were not available including: international brand cigarettes, fruit in 3 communities in India and 1 community in China, and vegetables in 7 communities from India.

### EPOCH 1 Reliability testing

Table 2 summarises the inter-rater reliability of environmental attributes for each section of EPOCH 1. Overall 24 of 38 variables had an ICC ≥0.75, 14 of 38 had an ICC between 0.40 and 0.74 and 0 of 38 had an ICC <0.40.

**Table 2:** Reliability testing of measurements from the EPOCH I instrument.

Environmental attributes		ICC	95% CI	Number of communities
*Community demographics*				
1	Cost of residential land	0.86	(0.80, 0.91)	59
2	Number of public transportation services (sum of yes responses to a list of 4 services)	0.93	(0.90, 0.95)	93
3	Maximum daily frequency of public transportation (6 categories)	0.94	(0.92, 0.95)	92
4	Number of types of public services/education facilities (sum of yes responses to list of 5 services)	0.86	(0.81, 0.90)	93
5	Number of types of community infrastructure (sum of yes responses to a list of 5 facilities)	0.93	(0.90, 0.95)	93
6	Number of types of community health facilities (sum of yes responses to a list of 7 facilities)	0.87	(0.83, 0.91)	93
7	Sidewalk completeness and quality score (scale of 0 to 8)	0.94	(0.91, 0.96)	93
*Community observation walk*				
1	Number of tobacco advertisements	0.67	(0.57, 0.75)	93
2	Number of signs prohibiting smoking	0.97	(0.96, 0.98)	93
3	Number of health promotion advertisements	0.54	(0.42, 0.65)	93
4	Number of snack food advertisements	0.79	(0.72, 0.85)	93
5	Number of sugary drink advertisements	0.88	(0.83, 0.91)	93
6	Number of alcoholic drink advertisements	0.87	(0.82, 0.91)	93
7	Number of places to buy cigarettes	0.80	(0.73, 0.85)	93
8	Number of places to buy snack foods	0.86	(0.81, 0.90)	93
9	Number of stores selling food	0.88	(0.84, 0.92)	93
10	Number of places to buy alcohol	0.69	(0.60, 0.77)	93
11	Number of restaurants	0.95	(0.93, 0.96)	93
12	Number of parks and street trees	0.80	(0.74, 0.86)	93
*Tobacco store assessment*				
1	In-store tobacco advertisements (yes/no)	0.83	(0.77, 0.88)	93
2	In-store smoking cessation promotion (yes/no)	0.71	(0.62, 0.79)	93
3	Number of tobacco brands	0.67	(0.57, 0.76)	93
4	Number of sizes of cigarette packs available (5 categories)	0.73	(0.64, 0.80)	93
5	Price of cheapest pack of cigarettes	0.96	(0.95, 0.98)	93
6	Price of Marlboro or other international brand	0.97	(0.95, 0.98)	68
7	Number of health warnings on cigarette packs	0.64	(0.52, 0.73)	93
*Grocery store assessment*				
1	Point of sale unhealthy food advertising (yes/no)	0.66	(0.56, 0.75)	93
2	Point of sale healthy food advertising (yes/no)	0.62	(0.52, 0.72)	93
3	Fruit and vegetable display quality (scale 1 to 7)	0.69	(0.60, 0.77)	93
4	Number of types of fruits available (checklist of 48 types)	0.86	(0.80, 0.90)	93
5	Number of types of vegetables available (checklist of 59 types)	0.90	(0.86, 0.93)	93
6	Price of fruit	0.83	(0.77, 0.88)	89
7	Price of vegetables	0.91	(0.87, 0.93)	86
8	Price of other products	0.86	(0.81, 0.90)	93
*Restaurant assessment*				
1	Healthy menu options (yes/no)	0.50	(0.37, 0.62)	88
2	Main salad or vegetables dish (yes/no)	0.58	(0.47, 0.69)	88
3	Buffet service (yes/no)	0.64	(0.54, 0.74)	88
4	Option to increase portion size (yes/no)	0.49	(0.37, 0.61)	88

**Note 1:** ***Public services/education facilities*** is the number of facilities from a list of 6: primary/secondary school, university/technical college, post office, police station, government building, public park. ***Community infrastructure*** is the number of characteristics from a list of 5: paved roads, traffic lights, street lights, internet and highway. Similarly ***Community health facilities*** are the number of characteristics from a list of 6: Public nurse-only clinic, Public medical clinic, Private medical clinic, Public hospital, Private hospital, Pharmacy that sells medications.

**Note 2:** The low numbers of communities for: ‘cost of residential land’ was because this data was not able to be obtained in many communities in Canada; for ‘Price of Marlboro’ and Restaurant variables was mainly because International brand cigarettes were not available in some rural Indian communities and some rural communities also did not have restaurants.

#### Reliability across sub-groups of communities

Findings were similar across urban and rural communities with 63% and 71% of items, respectively, having excellent reliability (Table 3). As the instrument was developed in Canada we compared reliability in Canadian communities with others. 71% of Canadian communities had excellent reliability compared with 61% of other communities. We also examined whether findings were similar in China, India and South America (Colombia and Brazil) and found higher levels of reliability in India and poorer levels in South America (Table 3).

**Table 3:** Reliability by region of EPOCH 1 measures: Number and proportion (%) of all items with ICC in the following ranges (38 items in total).

Grouping	Number of communities	Items with ICC <0.4		Items with ICC 0.4 to 0.75		Items with ICC >0.75	
		N	%	N	%	N	%
**All communities**	93	0	0	14	36.8	24	63.2
Urban	56	0	0	14	36.8	24	63.2
Rural	37	0	0	11	28.9	27	71.1
Canada	39	0	0	11	21.1	27	71.1
Other countries	54	0	0	15	42.1	23	60.5
Brazil/Colombia	20	8	21.1	14	36.8	16	42.1
India	15	2*	5.3	1	2.6	35	92.1
China	19	1	2.6	20	52.6	17	44.7

*Intraclass Correlation Coefficient (ICC) for these two items was not able to be calculated as was equal to zero for the majority of communities in India.

#### Item variability: (Appendix S4)

In China one item – ‘sizes of cigarette packs available’ had poor reliability. For this question observers had to visit a store that sold cigarettes and record the different sizes of cigarette packs available; in China many outlets sell cigarettes and the availability of different cigarette pack sizes in any two or more outlets varied. For example smaller vendor stalls sell smaller packs or single cigarettes.

In India, the reliability coefficient could not be calculated for two items. These were ‘signs prohibiting smoking’ and ‘in-store smoking cessation promotion’. For the first variable, the majority of the communities reported this as zero, with only two communities identifying one sign that prohibited smoking. For the second variable, the majority of communities reported zero while two communities reported one.

In Brazil/Colombia 8 items had poor reliability. One item was the ‘Number of health warnings on cigarettes’. The poor reliability for this variable appeared to be for two reasons. First, there was a misunderstanding regarding whether this question asked about the number of health warning labels or the number of different types of labels. That is, if there were identical health warning labels on the front and back of a pack this should have been counted as 2 labels and not 1. This misclassification also caused the lower reliability recorded in Canada. We identified this problem after data collection was completed. The second issue was true variability in number of health warnings on packs. In Canada, cigarette packs generally have the same number of health warnings, however in Brazil and Colombia there was true variability across cigarette packs. Five of the eight variables with poor reliability were measurements of numbers of advertisements or health promotion signs (‘signs prohibiting smoking’, health promotion advertisements’, ‘alcoholic drink advertisements’). Some observers identified many more advertisements than others. The 2 other variables with poor reliability were, ‘Healthy menu options in restaurants’ and ‘Main salad or vegetarian dish options in restaurants’. This seemed to be mainly due to observers attending different restaurants and these measures were not similar across different restaurants.

### EPOCH 2 administration

Researchers reported EPOCH 2 took between 10 and 20 minutes to administer and that the majority of questions were well understood with only occasional additional clarification being required. The variation that occurred across groups met expectations, for example few participants from Canada reported observing smokers smoking in public places in the last 6 months but in comparison many more participants from outside of Canada reported observing smoking in public places. Junk food advertising was prevalent through the different types of media in Canada but less prevalent in China and India. A large percentage of participants were aware of tobacco control policies in Canada compared to other countries. In India and China awareness of tobacco control policies was poorest in rural areas. Corresponding to this pattern, knowledge of the harms of smoking was greater in Canada and very low in India and China (Table 4).

**Table 4:** Participant observations and perceptions of their community environment – responses to EPOCH 2.

	Canada		China/India		Colombia/Brazil		All countries	
	Urban	Rural	Urban	Rural	Urban	Rural	Urban	Rural
Variable	Mean (SD) or %	Mean (SD) or %	Mean (SD) or %	Mean (SD) or %	Mean (SD) or %	Mean (SD) or %	Mean (SD) or %	Mean (SD) or %
***Participant observations***								
Proportion reporting observing smokers smoking in public places^1^	5%	2%	48%	46%	38%	53%	28%	36%
Mean number of types of media where Tobacco advertisements seen (total 7 types)	1.4 (0.5)	1.2 (0.5)	1.4 (0.5)	0.8 (0.5)	3.4 (1.6)	2.7 (1.3)	1.9 (1.2)	1.4 (1.1)
Mean number of types of media where Junk food advertisements seen (total 5 types)	3.4 (0.5)	3.5 (0.7)	1.9 (0.8)	0.7 (0.5)	2.8 (0.8)	2.0 (0.7)	2.7 (1.0)	1.8 (1.3)
Mean number of types of media where Healthy food advertisements seen (total 5 types)	3.0 (0.3)	2.9 (0.7)	2.0 (0.9)	0.9 (0.6)	2.2 (0.6)	1.7 (0.6)	2.5 (0.8)	1.6 (1.0)
***Participant opinions***								
Proportion reporting intolerance toindoor smoking	22%	9%	26%	30%	21%	26%	23%	23%
Proportion reporting disapproval of youth smoking	73%	75%	87%	96%	61%	59%	76%	80%
Proportion reporting disapproval of adult smoking	46%	45%	39%	84%	40%	35%	42%	60%
***Participant awareness***								
Proportion reporting awareness of bans on smoking in public	93%	88%	44%	25%	45%	42%	64%	47%
Proportion reporting awareness of bans on tobacco advertising	81%	82%	41%	20%	42%	34%	58%	41%
Proportion reporting awareness of laws on health warnings	94%	96%	55%	37%	56%	53%	71%	57%
Proportion reporting awareness of bans on youth smoking	67%	61%	41%	17%	25%	30%	48%	33%
Proportion reporting awareness of laws on food/drink labelling	82%	71%	28%	15%	43%	29%	53%	34%
***Participant knowledge***								
Dietary causes of CVD^2^	31%	23%	16%	11%	21%	18%	23%	16%
Smoking causes diseases^3^	17%	24%	2%	7%	10%	7%	10%	12%

1. Percent of participants that reported seeing smokers smoke anywhere in the grounds in one or more of the following public places: hospital, trains/bus or train/bus stations, out-of home eating venues (restaurants, cafes or bars), indoor areas of workplace.

2. Percent of participants that respond correctly to all 10 questions regarding dietary causes of CVD.

3. Percent of participants that respond correctly to all 8 questions regarding the diseases associated with smoking and second-hand smoke exposure.

## Discussion

Our investigation indicates that the collection of community-level information using the EPOCH instruments was feasible and, for many variables, direct community observation had high inter-observer reliability in communities in the 5 countries studied. There are no previous published reports to our knowledge of instruments that profile communities using a wide range of environmental factors influencing cardiovascular risk factors. Very few community profiling instruments have been examined for reliability and validity. An additional unique strength to our instrument is that it is suitable for use in large-scale epidemiological studies in countries at different levels of economic development and urbanisation.

As we have noted above, the majority of existing environmental assessment instruments assess single behavioural risk factors such as physical activity[2], [3], [4] while Raudenbush's “systematic social observation” work, which also uses community assessment, is restricted to the “social environment of the community”.[24] In some cases reliability has been assessed, but this has mainly been limited to assessment of inter-observer reliability. Brownson and colleagues found that measures of physical disorder and safety, which are often scored using a subjective measure or Likert scale, tend to be less reliable compared to objective measures such as land use and physical street characteristics.[6] Few studies have evaluated the validity of environmental measures and virtually no instruments have been evaluated across communities in high- middle- and low-income settings. The only exception that we are aware of was a simple perceived measure of how environmental attributes may affect physical activity in adults in 11 countries including China, Brazil, and Colombia, for which test-retest reliability was examined.[18]

Given the growing evidence that environmental factors are related to a variety of cardiovascular risk factors, there is an urgent need for an instrument that can reliably quantify environmental factors in diverse communities. This is further supported by the rapid environmental transition that many low and middle income countries are experiencing which will likely impact chronic disease rates in those countries.

Unlike previous tools, our instrument assesses a composite of environmental factors, which is important from a public policy perspective as such factors influence several health-related behaviours. It has undergone numerous iterations to arrive at a set of measures that can feasibly be collected by research assistants following basic training in diverse communities. The reliability of the items measured by direct observation (EPOCH 1) is generally high. The instrument performed least well in Brazil and Colombia where 8 of the 38 items had low reliability. This seemed to be due mainly to: 1) measures being truly variable, for example cigarette packs did not have a uniform number of health warnings on them; 2) observers having different understandings of definitions, leading to identification of different numbers of advertisements. This may, however, reflect the lack of in-person training in the Colombian and Brazilian centres. It may be that improved face-to-face training would resolve this.

The qualitative feedback from observers was important in refining the instrument. Thus, some observers reported including pubs/restaurants that sell alcohol in ‘Places to buy alcohol’ and others included only specialty stores selling alcohol. Different assessments of point-of-sale advertising of healthy/unhealthy foods were due to confusion about the definition of ‘point of sale’. Feedback from observers indicated that some only responded yes to ‘point of sale’ advertising if the advertisement was beside the cashier, while others responded yes if they observed advertising at any place at the front of the store. Observers also noted that identification of advertisements seems to improve as observers ‘learn’ where to look. We have subsequently improved our EPOCH manual and training materials to address these. We also now require trainers and auditors do at least one community assessment together to discuss observations, definitions and methods prior to actual data collection.

This study has some limitations. It was conducted in a convenience sample of communities in a small number of countries. We would encourage other groups that may be interested in using this instrument to assess instrument reliability in their setting prior to use. Practice in using the instrument is likely to improve reliability. We did not evaluate intra-observer differences (i.e. the differences between repeat assessments by the same person on the same day of a community). It was expected that these would be very minimal due to the nature of the measures. We expected the main source of measurement error to be inter-observer differences. We also did not assess the test-retest reliability of the EPOCH 2 instrument of perceptions of environments and policies. The measures of the alcohol environment are limited to availability of places to buy alcohol and advertising in the community and omit measures of alcohol-related policy.

### Conclusions

This report describes the design and development of an instrument to collect information about the community environment from a variety of settings and shows data collection with the EPOCH instrument is feasible and direct observation of community measures reliable. The EPOCH instrument will further research in the field of environmental determinants by making possible the examination of the nature and strength of the relationship between community-level factors and individual health for population studies from a broad range of settings.

## Supporting Information

Appendix S1 EPOCH 1 instrument: Version August 21, 2008
(0.04 MB PDF)

Appendix S2 EPOCH 2 instrument: version September 4, 2008
(0.02 MB PDF)

Appendix S3 EPOCH manual: version September 8, 2008
(0.28 MB PDF)

Appendix S4 Reliability testing by country
(0.25 MB DOC)
