# Supplementary material for: Environmental Profile of a Community's Health (EPOCH): An Instrument to Measure Environmental Determinants of Cardiovascular Health in Five Countries
Source: PLoS One. 2010 Dec 10;5(12):e14294. doi: 10.1371/journal.pone.0014294 (PMC3000812; doi:10.1371/journal.pone.0014294)
Supplement: Appendix S1 — EPOCH 1 instrument: Version August 21, 2008 (0.04 MB PDF) [file pone.0014294.s001.pdf]

Community ID

☐☐ ☐☐☐
Centre # *Community#*

Date: ☐☐ ☐☐ ☐☐☐☐
day *month* *year*

1. Was a map of the community obtained?

☐ No ☐ Yes, map of route attached

a) Additional measures taken to define the community area:

____

____

____

2. Which statement best describes the arrangement of your community (check one only)**:**

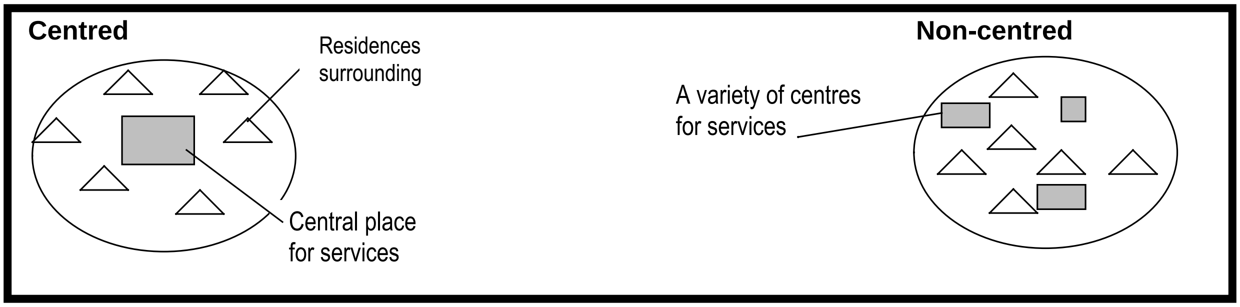

☐ Centred ☐ Non-centred ☐ Other, please describe____________________

Community Demographics (Please answer all prices in local currency)

3. Community name/s ____________________________________

4. Postal code/s of area ☐☐☐☐☐☐☐☐☐☐

5. Centre name ____________________________________

6. Cost per unit area of residential land ☐☐☐☐☐☐☐☐☐☐ ________ Unit of measure ________ Local currency

7. Is this community connected to other towns/cities with a network of transport e.g. bus, train or tram network?

☐ No, there are no regular services **(Go to Question #8)**

☐ Yes, there is a regular service

a) What types of services are there? (mark all that apply)

☐ Train ☐ Bus ☐ Shared Taxi ☐ Other, please specify____________________

Community ID

☐	☐	☐	☐	☐

Centre # *Community#*

b) What is the maximum frequency per day of any regular services? (Mark one box only)

- ☐ 2 or more times per hour (20 or more times per day)
- ☐ Hourly (10 to 19 times per day)
- ☐ 5 to 9 times per day
- ☐ 2 to 4 times per day
- ☐ Daily
- ☐ Less than once a day

8. Do the following facilities exist in this community?

No	Yes	
☐	☐	Supermarket where you can buy food supplies
☐	☐	Free Market **(China only)**
☐	☐	General store/ Convenience store
☐	☐	Market store (e.g. bakery, butcher, fruit market)
☐	☐	Store that sells tobacco/ cigarettes
☐	☐	Restaurant/ cafe/ fast-food outlet - place where one can buy food and **sit down and eat** it in an establishment outside the home
☐	☐	Take out store/ Street store/ coffee or food cart/ Tea shop/ Food stand - place where you can buy food - but there is **no place to sit down**.
☐	☐	Vending machine - where you can buy snack foods/ soft drinks
☐	☐	Vending machine - where you can buy cigarettes
☐	☐	Primary or Secondary School
☐	☐	College/ University/ Post-secondary Technical college
☐	☐	Post office
☐	☐	Police station
☐	☐	Government building accessible to community (e.g. community centre, library)
☐	☐	Public Park/ recreational area/ gardens
☐	☐	Paved roads
☐	☐	Electrical street lighting
☐	☐	Internet access enabled
☐	☐	Traffic lights
☐	☐	Factory
☐	☐	A highway - where cars can exceed speeds of 50km/ hour

Community ID

☐☐ ☐☐☐

Centre # *Community#*

9. Do the following health care facilities exist in this community?
(Public refers to government run)

No	Yes	
☐	☐	Public nurse-only clinic
☐	☐	Public medical clinic
☐	☐	Private medical clinic
☐	☐	Public hospital (Government hospital)
☐	☐	Public sector hospital (Hospital for employees) **(India only)**
☐	☐	Private hospital
☐	☐	Chemist/ pharmacy that sells medications

10. Is this a rural community?

☐ No, **(Go to Question #13, Next section Community Observation Walk)**

☐ Yes, **(Go to Question #11, Additional questions for rural communities)**

Additional questions for Rural Communities

11. What is the estimated distance from the centre of this community area to each of the following (in Kilometers)?
Note: If a railway station/ other transport/ tarred roads exist **within** the boundaries of this community, put zero kilometers.

a) Nearest city/ major urban centre ☐☐☐☐ . ☐ km

b) Nearest national state highway ☐☐☐☐ . ☐ km

c) Nearest long distance bus station ☐☐☐☐ . ☐ km

d) Nearest railway station with passenger trains stopping at least twice per day ☐☐☐☐ . ☐ km

12. What is the approximate travel time (in minutes) to travel from the centre of this communtiy to the following (during regular working hours)?

a) Nearest city/ major urban centre on public transport ☐☐☐☐ minutes

b) Nearest city/ major urban centre in a motorized vehicle ☐☐☐☐ minutes

Community ID

☐☐ ☐☐☐

Centre # *Community#*

COMMUNITY OBSERVATION WALK

13. Record today's date: ☐☐ ☐☐ ☐☐☐☐ (*day* *month* *year*) **Start Time** ☐☐ : ☐☐ (00:00-23:59)

14. The Start point:

a) Which description best describes the central <u>start point</u> (Mark one only)

- ☐ Central busy intersection
- ☐ Market
- ☐ Central train station
- ☐ Shopping mall
- ☐ Central bus station
- ☐ School
- ☐ Post office
- ☐ Other - please specify ____________________
- ☐ Supermarket or general store/ convenience store

b) What is the address of the start point?

i) Street No.: ☐☐☐☐☐

ii) Street Name: ____________________

iii) Cross street: ____________________

iv) Latitude: ☐☐ . ☐☐☐☐☐ ☐ (Enter N for North or S for South)

v) Longitude: ☐☐ . ☐☐☐☐☐ ☐ (Enter E for East or W for West)

vi) Total distance: walked (meters) ☐☐☐☐☐

15. Was a pre-planned route obtained? ☐ No ☐ Yes, route drawn or attached to page 5

16. Sidewalk completeness and quality

a) Please indicate which best describes the completeness of the sidewalk on your route. (Mark one only)

- ☐ No sidewalk
- ☐ Partial sidewalk (sections with no sidewalk)
- ☐ Complete sidewalk on one side
- ☐ Complete sidewalk on both sides

b) Give a rating between 1 and 4 for the quality of the sidewalk on your route. ☐
(Score 1 - if poorly maintained, score, 4 if well maintained)

Community ID

Centre # *Community#*

Pre-planned route:

Observational walk

Follow the instructions exactly on community observation to assess this community environment for advertisements and availabiltiy of local shops/public places and document in the following tables.
Walk down the street for approximately 300 to 500 meters then cross the road and return walking back on the other side of the street returning to the spot opposite from where you started on the other side.
As you walk, look around you for advertising (e.g. billboards, posters, signs on shops, walls, bus stop shelters, advertisements on buses/cars etc.) and the types of shops. Each time you see one of the advertisements types or shops of interest listed below, mark a check or line in the Tally column.
At the end of your walk, total the tally columns for each row.

Photographic assessment

A series of photos will assist us in qualitatively comparing neighbourhoods and will also assist communities in identifying the locations researchers have assessed. We suggest the following photographs to be included in your assessment. Please carefully label all your photos with the date and community ID number.

a. Photo of the street scene in each direction from the **start point** (a minimum of 4 photos)
b. Examples of **advertisements** that are classified in question 17a (please include one photo of each category of advertisement that is identified on your community observation walk
c. Outside/Front of shop of the **tobacco shop** visited
d. Outside/Front of shop of the **grocery store** visited
e. Photo of **fruit and vegetable display** in the shop or in the stall (a minimum of 2 photos, 1 of fruits and one of vegetables)

Community ID

Centre # *Community#*

17a) **Advertisements**	**Tally of Advertisements**	**Total**
i) Cigarette/tobacco product		
ii) Signs that prohibit smoking		
iii) Health promotion (smoking cessation)		
iv) Health promotion (alcohol cessation)		
v) Snack food		
vi) Sugary drink (eg Coke, juices, sports drink)		
vii) Non-commercial Health promo (diet)		
viii) Commercial Health promo (diet)		
ix) Non-commercial Health promo (Phys Act)		
x) Commercial Health promo (Phys Act)		
xi) Alcoholic drinks		
**17b) Shops**		
i) Vending machines (cigarettes)		
ii) Vendors/street stands (cigarettes)		
iii) Convenience /general store (cigarettes)		
iv) Vending machines (snack foods)		
v) Vending machines (sweet drinks)		
vi) Vendors/street stands/snack food shops		
vii) Convenience/general store (no cigarettes)		
viii) Supermarket		
viii) Free market (China)		
ix) Fruit & vegetable store/market		
x) Butcher/meat store/market store		
xi) Bakery		
xii) Deli/other speciality food store		
xiii) Alcohol speciality stores		
xiv) Fast food restaurants		
xv) Cafes/fast casual restaurants		
xvi) Pubs/bars		
xvii) Sit down restaurants		
**17c) Public Places**		
i) For recreation/ physical activity		
ii) Street trees/street flower beds		

Community ID

☐☐ ☐☐☐

Centre # *Community#*

TOBACCO STORE ASSESSMENT

18. Identify an outlet that sells cigarettes/smoked tobacco on your walk. If none available, walk to nearest place that sells cigarettes. If more than one available, visit the first store identified.

What is the street location of this spot and nearest main cross street? (Take a photo of the front of the tobacco store)

a) Distance from start point: (Meters) ☐☐☐☐☐

b) Street number: ☐☐☐☐☐

c) Street name: ______________________

d) Main cross street: ______________________

19. Referring to the above store, do you see any of the following:

No	Yes	
☐	☐	Point-of-sale tobacco advertising
☐	☐	Cigarettes/smoked tobacco openly displayed (can you see cigarettes without requesting to buy them)
☐	☐	Signs that prohibit smoking in the store
☐	☐	Signs/information regarding the harmful effects of smoking visible on entering the store/ approaching the counter

20. How many brands of cigarettes are sold in this store? ☐☐☐

20a) How many brands of beedis are sold in this store?(India) ☐☐☐

20b) How many brands of chewing tobacco are sold in this store?(India) ☐☐☐

21. In what size packets are cigarettes sold in this store? (Mark all that apply)

☐ Singles Units ☐ 2-10/pack ☐ 11-19/pack ☐ 20 - 24/pack ☐ 25 or more/pack

22. Record the cost of a pack of the cheapest cigarettes and a pack of Marlboro. If no Marlboro, use other international brand. **If there is a variety of pack sizes, record the cost, in local currency, of a pack of 20.** (or pack nearest to 20 units)

	Brand	Price	Number in Pack
a) Cheapest pack	______________	☐☐☐☐.☐☐	☐☐
b) Marlboro (or other international brand)	______________	☐☐☐☐.☐☐	☐☐

23. Buy the "local brand of cigarettes" priced above and send packet back to project office.

a) Is there a health warning on the packet? ☐ No ☐ Yes, answer 23b and c

b) What is the location of the warning on the packet? (Check all that apply)

☐ Front ☐ Back ☐ Side ☐ Top ☐ Bottom

c) Transcribe the warning and translate into English here:

____

____

Community ID

☐☐ ☐☐☐

Centre # *Community#*

GROCERY STORE ASSESSMENT

24. Which best describes the food purchasing environment in this community? (Mark one only)

- ☐ Supermarket- where all groceries are sold under one roof
- ☐ Cluster of small stores/market stores clustered together in a defined permanent market area
- ☐ Discrete stores that are permanent and specialize in separate groceries (eg.butcher,bakery etc)
- ☐ Combination of discrete permanent stores and non-permanent/seasonal street vendors
- ☐ Multiple street vendors not housed in a building or permanent store

25. Mark the type of store or stores you need to visit to buy the items listed in Q28. (Mark all that apply)

- ☐ Supermarket
- ☐ General store
- ☐ Fruit and vegetable store
- ☐ Cluster of street vendors or market stores
- ☐ Butcher
- ☐ Baker
- ☐ Other food store ____________________
- ☐ Other food store ____________________
- ☐ Other food store ____________________

26. Visit the store/ supermarket to obtain food prices. What is the location of the grocery store?
(Photograph the front of the store)

a) Distance from the start point: ☐☐☐☐☐
(Meters)

b) Street number: ☐☐☐☐☐

c) Street name: ____________________

d) Main cross street: ____________________

27. Referring to this store, assess for the presence of the following:
(If more than one refer to closest store)

No	Yes	
☐	☐	Advertisements at the point of sale for snack foods
☐	☐	Advertisements at the point of sale for sugary drinks
☐	☐	Advertisements at the point of sale for fruits and/or vegetables

Community ID

Centre #	Community#

28. Record the cost of the list of groceries below. (Please answer all prices in local currency)
Calculate the cost of one egg based on a pack of about 12 medium sized eggs.
If specified item type is not available, cost the cheapest type available and note the type in the space provided.

Fruit or Vegetable	Cost	Item Type Specification
a) 1kg Apples	☐☐☐☐.☐☐	☐ Red delicious ☐ Other ______
b) 1kg Oranges	☐☐☐☐.☐☐	☐ Navel ☐ Other ______
c) 1kg Bananas	☐☐☐☐.☐☐	
d) 1kg Pear (China Only)	☐☐☐☐.☐☐	☐ Duck pear ☐ Other ______
e) 1kg Carrots	☐☐☐☐.☐☐	
f) 1kg Tomatoes	☐☐☐☐.☐☐	☐ Loose ☐ Other ______
g) 1 medium sized cabbage	☐☐☐☐.☐☐	

Food Item	Cost	Additional information
h) 1 litre of regular milk	☐☐☐☐.☐☐	☐ Shop brand ☐ Other ______
i) 1 litre of low fat (1%) milk	☐☐☐☐.☐☐	☐ Shop brand ☐ Other ______
j) 1 loaf of white bread	☐☐☐☐.☐☐	☐ Shop brand ☐ Other ______
k) 1kg white rice	☐☐☐☐.☐☐	☐ Shop brand ☐ Other ______
l) 1kg chicken drumsticks with skin	☐☐☐☐.☐☐	
m) 1 kg pork meat (China only)	☐☐☐☐.☐☐	☐ Shop brand ☐ Other ______
n) 1 egg	☐☐☐☐.☐☐	☐ Shop brand ☐ Other ______
o) 1 can/bottle of cola	☐☐☐☐.☐☐	Volume of cola ☐☐☐☐☐ ______ unit of measure
p) 1 small chocolate bar (50 to 150 grams)	☐☐☐☐.☐☐	☐ Hersheys ☐ Other ______<br>Weight of chocolate bar ☐☐☐☐☐ ______ unit of measure

Community ID

☐☐ Centre # ☐☐☐ Community#

29. What types of fruit were available in this store/ group of stores you have attended? (Check all that apply)

☐ Apple	☐ Orange	☐ Lychee (East Asia)
☐ Apricots	☐ Papaya/ Paw paw	☐ Pomelo/Shddock (East Asia)
☐ Avocado	☐ Peach	☐ Shan Zhu (East Asia)
☐ Banana	☐ Pear	☐ Cape gooseberry (S. America)
☐ Cantalope	☐ Persimmon	☐ Coconut (S. America)
☐ Cherries	☐ Pineapple	☐ Curuba (S. America)
☐ Dates (fresh)	☐ Plum	☐ Granadilla (S. America)
☐ Fig	☐ Pumpkin	☐ Lulo (S. America)
☐ Grapefruit	☐ Raspberries	☐ Passion fruit (S. America)
☐ Grapes	☐ Strawberries	☐ Soursop (S. America)
☐ Guava	☐ Other berries	☐ Tamarillo (S. America)
☐ Honeydew Melon	☐ Watermelon	☐ Tamarind (S. America)
☐ Kiwi	☐ Custard Apple (East Asia)	☐ Zapote (S. America)
☐ Lemon/ Lime	☐ Fire Dragon fruit (East Asia)	☐ Jamun (South Asia)
☐ Mango	☐ Jackfruit (East Asia)	☐ Pomegranate (South Asia)
☐ Nectarine	☐ Longan (East Asia)	☐ Sweet Lime (South Asia)

30. Answer the following questions with regard to the <u>fruit display</u> in the store (or first store if multiple) that you visited to price fruit. (Photograph the fruit display to capture quality of fruit avaiable in the shop)

a) Are the fruits in this store easily visible from the outside of the store? ☐ No ☐ Yes

b) Do more than 3 kinds of fruits appear to be damaged (bruised, rotten, or of poor quality)? ☐ No ☐ Yes

c) Have more than 3 kinds of fruits in this store been specially packaged, wrapped or boxed for sale? ☐ No ☐ Yes

Community ID

☐☐ ☐☐☐

Centre # *Community#*

31. What types of vegetables were available in this store/ group of stores you have attended? (Check all that apply)

☐ Alfalfa sprouts	☐ Kale	☐ Ipomoea (East Asia)
☐ Artichokes	☐ Leek	☐ Kalimeris indica (East Asia)
☐ Asparagus	☐ Lettuce	☐ Kohrabi leaf (East Asia)
☐ Beets	☐ Mushrooms	☐ Snow Pea/Holland beans (East Asia)
☐ Bok Choi	☐ Mustard Greens	☐ Sponge gourd, loofa (East Asia)
☐ Broad(Lima) beans	☐ Okra/lady finger	☐ Winter Melon (East Asia)
☐ Broccoli	☐ Onions	☐ Chard (S. Amercia)
☐ Brussel Sprouts	☐ Other Chinese green vegetables	☐ Kusk (S. America)
☐ Cabbage	☐ Parsnips	☐ Bitter gourd (South Asia)
☐ Carrots	☐ Peas	☐ Bottle gourd (South Asia)
☐ Capsicums/Peppers	☐ Radish	☐ Chow Chow (South Asia)
☐ Cauliflower	☐ Spinach	☐ Colacasia (South Asia)
☐ Celery	☐ Squash	☐ Drumstick (South Asia)
☐ Chinese cabbage	☐ Tomatoes	☐ Green leafy (South Asia)
☐ Collards	☐ Turnips	☐ Karamani (South Asia)
☐ Corn	☐ Zucchini	☐ Other sprouts (South Asia)
☐ Cucumber	☐ Bean sprouts -Mung (East Asia)	☐ Ridge gourd (South Asia)
☐ Eggplant/Brinjal	☐ Bean sprouts -Soy (East Asia)	☐ Snake gourd (South Asia)
☐ Gourd	☐ Chrysanthemum (East Asia)	☐ Ulam (Malaysia)
☐ Green beans	☐ Cowpea (East Asia)	

32. Answer the following questions with regard to the vegetable display in the store (or first store if multiple) that you visited to price vegetables. (Photograph the vegetable display to capture quality of fruit avaiable in the shop)

a) Are the vegetables in this store easily visible from the outside of the store? ☐ No ☐ Yes

b) Do more than 3 kinds of vegetables appear to be damaged (bruised, rotten, or of poor quality)? ☐ No ☐ Yes

c) Have more than 3 kinds of vegetables in this store been specially packaged, wrapped or boxed for sale? ☐ No ☐ Yes

Community ID

☐☐ ☐☐☐

Centre # *Community#*

33. Choose one only packaged snack food. Fill in the table below.

a) Brand name of item: ____________________

b) Description of food type: ____________________

c) Country where produced: ____________________

d) Cost of item: ☐☐☐☐.☐☐

34. Referring to the above item, does the label of this item have information on any of the following?

No	Yes	
☐	☐	Energy (Kcal or Kjoule)
☐	☐	Total Fat
☐	☐	Saturated fat
☐	☐	Trans fat

35. Referring to the above item, please write all the written information including name of product, health claims, ingredients and nutrition information.

____

____

____

____

____

____

____

____

____

____

____

____

Question #38 -

Sit down Restaurants - offers full table service by wait staff who take your order at the table, menu provided.

Bars/Pubs - sells a full range of alcoholic beverages, may have large quantity of alcohol advertisements

Fast casual restaurant - similar to fast-food in that it does not offer table service but promises somewhat higher quality of food and atmosphere. You may order and pay at a counter. Often food is brought to table.

Fast food restaurant - These are characterized by minimal service and by food that is supplied quickly after ordering. Food is commonly cooked in bulk in advance and kept hot, or reheated to order. Fast food is often finger food that can be eaten quickly without cutlery.

Outdoor food stall, with seating, selling pre-cooked or food cooked to order, with minimal or no facilities.

Community ID

☐☐ ☐☐☐

Centre # *Community#*

RESTAURANT ASSESSMENT

36. Identify a typical low cost sit-down restaurant (inside or in the street) using the first available option in the list below.

- ☐ Closest to the starting point within the streets immediately surrounding (1000 meter radius)
- ☐ Closest to supermarket/ grocery stores within the streets immediately surrounding (1000 meter radius)
- ☐ Other location ____________________

37. What is the location of this restaurant and nearest main cross street?

a) Distance from start point: (Meters) ☐☐☐☐☐

b) Street number: ☐☐☐☐☐

c) Street name: ____________________

d) Main cross street: ____________________

38. What type of restaurant is it? See facing page for descriptions (Mark one only)

☐ Sit down	☐ Fast food
☐ Bars/Pubs	☐ Outdoor food stall
☐ Fast casual	☐ Other, please specify ____________________

39. In the restaurant or restaurant area:

a) Do signs/ table information/menus highlight healthy menu options?	☐ No	☐ Yes
b) Does the menu include any main dish salads or vegetable dishes?	☐ No	☐ Yes
c) Is there a buffet sevice available in this restaurant?	☐ No	☐ Yes
d) Are there options in this restaurant to increase the portion size of your meal for a small price? (eg. all-you-can-eat/drink, super-size, jumbo)	☐ No	☐ Yes

Obtain a copy, transcribe or photograph the menu in this restaurant. Take this menu to the office and transcribe and translate each item into English.

40. Name of Interviewer: ________ ____________________ **End time:** ☐☐ : ☐☐
(please print) *First Initial* *Last Name* (00:00-23:59)
