# Supplementary material for: Environmental Profile of a Community's Health (EPOCH): An Instrument to Measure Environmental Determinants of Cardiovascular Health in Five Countries
Source: PLoS One. 2010 Dec 10;5(12):e14294. doi: 10.1371/journal.pone.0014294 (PMC3000812; doi:10.1371/journal.pone.0014294)
Supplement: Appendix S2 — EPOCH 2 instrument: version September 4, 2008 (0.02 MB PDF) [file pone.0014294.s002.pdf]

Subject ID

Centre #	Community#	Household #	Subject #

Subject Initials F M L

Community Tobacco Environment

Interviewer to read: **"In the following questions I'll be asking about where you have smoked or where you have seen smokers smoke in the last year. If you haven't been to these areas in the last year and can not report on your experience then you can say you are unsure."**

1. Which statement best describes the situation with smoking in acute care hospitals in your community *(or hospital nearest to your community that community members would use?)* (check one only)

- ☐ Smokers smoke anywhere in the hospital
- ☐ Smokers smoke only in certain areas (designated indoor smoking areas)
- ☐ Smokers only smoke outside
- ☐ There is no smoking anywhere on premises indoor or outdoor
- ☐ Don't know/ Unsure *(do not read this option out, only mark this option if the person states they are unsure)*

2. Which statement best describes the situation with smoking in trains/buses and train/bus stations in your community, *(or the nearest transport to your community that community members would use?)* (check one only)**:**

- ☐ Smokers smoke anywhere on trains/buses and at the station
- ☐ Smokers smoke only in certain areas of trains or buses (designated smoking carriages or sections)
- ☐ Smokers do not smoke anywhere on trains or buses but do smoke in the stations
- ☐ Smokers do not smoke anywhere on trains or buses or at the stations
- ☐ Don't know/ Unsure *(do not read this option out, only mark this option if the person states they are unsure)*

3. Which statement best describes the situation with smoking in out-of-home eating venues such as restaurants, cafes or bars in your community? (check one only)**:**

- ☐ Smokers smoke anywhere in eating venues
- ☐ Smokers smoke only in certain areas (designated indoor smoking areas)
- ☐ Smokers only smoke outside
- ☐ There is no smoking anywhere on premises indoor or outdoor
- ☐ Don't know/ Unsure *(do not read this option out, only mark this option if the person states they are unsure)*

4. Which of the following best describes smoking in your workplace? (check one only)

- ☐ Smoking is allowed in any indoor area
- ☐ Smoking is not allowed in any indoor area
- ☐ Smoking is allowed in some indoor areas
- ☐ There are no rules about smoking inside your workplace
- ☐ Not applicable (e.g. Do not work/ do not have a workplace)

Subject ID

Centre #	Community#	Household #	Subject #

Subject Initials F M L

5. **Which statement best describes the rules about smoking inside your home?** (check one only)

- ☐ Smoking is allowed in any indoor area
- ☐ Smoking is allowed in some indoor areas
- ☐ Smoking is not allowed in any indoor area
- ☐ There are no rules about smoking inside your home

6. For each of the following public places, please tell me if you think smoking should be allowed in
Outside refers to outside areas within the public places's grounds or at or near the entrances to the place.
(Mark only one for each)

	All indoor areas	Some designated indoor areas	Outside only	Not allowed outside or inside
a) Hospitals	☐	☐	☐	☐
b) Workplaces	☐	☐	☐	☐
c) Restaurants and cafes	☐	☐	☐	☐
d) Public library or art gallery	☐	☐	☐	☐
e) Public parks/ gardens	☐	☐	☐	☐

7. **In the last 6 months in your community have you seen any of the following?**

No	Yes	
☐	☐	Cigarette advertisements on posters (eg. billboards, pasted on walls, visible on sides of taxis, buses etc.)
☐	☐	Cigarette advertisements on television/ radio
☐	☐	Cigarette advertisements in movies at cinemas
☐	☐	Cigarette advertisements in newspapers/ magazines
☐	☐	Cigarette sponsorship of sporting, music, other events
☐	☐	Cigarette advertising on products such as umbrellas, ashtrays, clothing or any other product
☐	☐	Actors/ actresses smoking in films/ movies or TV shows

8. **In the last 6 months have you seen any advertisements/ articles/ programs regarding the importance of quitting smoking or regarding the health effects of smoking in any of the following types of media?**

No	Yes	
☐	☐	Television/ radio
☐	☐	Newspapers/ magazines
☐	☐	Posters (e.g. billboards, pasted on walls, visible on the sides of taxis, buses etc.)

9. **From your general observation, which statement best describes how easy it is for youth (teenagers/ children < 18yrs of age) to buy cigarettes/beedies in this community or in nearby stores used by this community?** (check one only)

- ☐ Can buy cigarettes in most (nearly all) outlets
- ☐ Can buy cigarettes in some outlets
- ☐ Can not buy cigarettes in any outlets
- ☐ Don't know/Unsure *(do not read this option out, only mark this option if the person states they are unsure)*

Subject ID

Centre #	Community#	Household #	Subject #
☐☐	☐☐☐	☐☐☐	☐☐

Subject Initials ☐☐☐ (F M L)

	No	Yes	Unsure
**10. Do you think society disapproves of children (<15yrs) smoking cigarettes?**	☐	☐	☐
**11. Do you think society disapproves of children (16-19) smoking cigarettes?**	☐	☐	☐
**12. Do you think society disapproves of women smoking cigarettes?**	☐	☐	☐
**13. Do you think society disapproves of men smoking cigarettes?**	☐	☐	☐
**<u>South Asia Only</u>**			
**10SA. Do you think society disapproves of young people smoking beedis?**	☐	☐	☐
**11SA. Do you think society disapproves of women smoking beedis?**	☐	☐	☐
**12SA. Do you think society disapproves of men smoking beedis?**	☐	☐	☐
**<u>Middle East Only</u>**			
**10ME. Do you think society disapproves of young people smoking waterpipes?**	☐	☐	☐
**11ME. Do you think society disapproves of women smoking waterpipes?**	☐	☐	☐
**12ME. Do you think society disapproves of men smoking waterpipes?**	☐	☐	☐
**14. Are there any support programs (government or non-governmental organization run programs or clinics, telephone quit-lines) that individuals in your community can access to help them stop smoking?**	☐	☐	☐
**15. Are you aware of any laws (national/ state or local government) that ban/ restrict smoking in public places?**	☐	☐	☐
**16. Are you aware of any laws (national/ state or local government) that ban/ restrict tobacco advertising?**	☐	☐	☐
**17. Are you aware of any laws (national/ state or local government) that mandate health warnings on cigarette packets?**	☐	☐	☐
**18. Are you aware of any laws (national/ state or local government) that prohibit smoking in youth?**	☐	☐	☐

19. I am going to read out a list of health effects and diseases that may or may not be caused by smoking cigarettes. Based on what you know or believe, does smoking cause any of the following?

	No	Yes	Unsure
a) Heart disease in smokers	☐	☐	☐
b) Arthritis in smokers	☐	☐	☐
c) Stroke in smokers	☐	☐	☐
d) Diabetes in smokers	☐	☐	☐
e) Lung cancer in smokers	☐	☐	☐
f) Lung cancer in non-smokers from exposure to other people smoking	☐	☐	☐
g) Asthma in non-smokers from exposure to other people smoking	☐	☐	☐
h) Heart disease in non-smokers from exposure to other people smoking	☐	☐	☐

Subject ID

☐☐ Centre # ☐☐☐ Community# ☐☐☐ Household # ☐☐ Subject #

Subject Initials ☐☐☐ F M L

For Current Smokers:

20. During the last 12 months did you receive advice from a doctor or health professional to quit smoking?

☐ **No** ☐ **Yes**

21. The last time you bought cigarettes, how many cigarettes did you buy?

a) Number of packs ☐☐☐

b) Number of cigarettes per pack ☐☐☐

c) If not in packs, number of loose cigarettes ☐☐☐

d) Date of purchase *day* ☐☐ *month* ☐☐ *year* ☐☐☐☐

e) Price paid for total purchase ☐☐☐☐ . ☐☐ Local currency ____________________

Community Nutrition/ Physical Activity Environment

22. In the last 6 months have you seen any junk food/ unhealthy snack food advertisements in the following media in your community? *(Junk food/ unhealthy snack food generally are snack foods of low nutritional value e.g. (give country-specific examples of snacks)*

No	Yes	
☐	☐	On Posters (e.g. Billboards, pasted or painted on walls, visible on the sides of taxis, buses etc.)
☐	☐	TV/ radio
☐	☐	Newspapers/ magazines
☐	☐	Sponsorship of sporting, music or other cultural events
☐	☐	Products such as umbrellas, ashtrays, clothing

23. In the last 6 months have you seen any health food/fresh fruit/fresh vegetable advertisements in the following media in your community?

No	Yes	
☐	☐	On Posters (e.g. Billboards, pasted or painted on walls, visible on the sides of taxis, buses etc.)
☐	☐	TV/ radio
☐	☐	Newspapers/ magazines
☐	☐	Sponsorship of sporting, music or other cultural events
☐	☐	Products such as umbrellas, ashtrays, clothing

24. In the last 6 months have you seen any advertisements/programs/articles promoting the importance of good diets to maintaining good health in any of the following media?

No	Yes	
☐	☐	Television/ radio
☐	☐	Newspapers/ magazines
☐	☐	On posters (e.g. Billboards, pasted on walls, visible on the sides of taxis, buses etc.)

Subject ID

☐☐	☐☐☐	☐☐☐	☐☐
Centre #	*Community#*	*Household #*	*Subject #*

Subject Initials ☐☐☐
F M L

25. In some countries, processed foods *(packaged food usually made or specially packaged in factories to last longer than fresh foods)* **carry a Nutrient label listing amounts or percentages of different types of nutrients (e.g. fat, saturated fat, salt, protein, sugar etc.) contained in the food. Have you seen Nutrient labelling on processed foods sold in stores in your community?** (check one only)

- ☐ Most foods have Nutrient labelling
- ☐ A few processed foods have nutrient labelling
- ☐ There is no nutrient labelling on processed foods
- ☐ Unsure/ Don’t know *(do not read this option out, only mark this option if the person states they are unsure)*
- ☐ Unsure as can not read

26. Which of the following actions may prevent/stop a person from getting a heart attack or stroke? Based on what you know or believe indicate no, yes or unsure for each statement.

	**No**	**Yes**	**Unsure**
a) Doing more exerciase	☐	☐	☐
b) Eating more fruit	☐	☐	☐
c) Eating more green vegetables	☐	☐	☐
d) Eating more meat	☐	☐	☐
e) Drinking more coffee	☐	☐	☐
f) Eating more dairy products (e.g. milk, cheese)	☐	☐	☐
g) Eating more fish	☐	☐	☐
h) Smoking	☐	☐	☐
i) Reducing fat in meals	☐	☐	☐
j) Reducing salt in meals	☐	☐	☐
k) Gaining weight	☐	☐	☐
**27. Are you aware of any official dietary guidelines on healthy foods/ diets?**	☐	☐	☐
**28. Are you aware of any laws (national/state or local government) that mandate nutrient labelling on foods/ beverages?**	☐	☐	☐
**29. Are you aware of any laws (national/state or local government) that subsidize or lower tax paid on fresh fruits and/or vegetables such that they are cheaper to buy?**	☐	☐	☐

Community Social Environment

30. Would adults in this community tell children, who are not their own children, to stop smoking? *(check one only)*

- ☐ This is common for people in my neighbourhood
- ☐ Some adults would do this in our neighbourhood
- ☐ Infrequently, but it may happen
- ☐ This would not occur in our neighbourhood

35f) Occupation Codes

Group 1: Legislators, senior officials and managers

Legislators and senior officials
Corporate managers
General managers
Businessman

Group 2: Professionals

Physical, mathematical and engineering science professionals
Life science and health professionals
Teaching professionals
Other professionals

Group 3: Technicians and associate professionals

Physical, mathematical and engineering-science associate professionals/technicians
Life science and health associate professionals/technicians
Teaching associate professionals/technicians
Other associate professionals/technicians

Group 4: Clerks

Clerks
Customer service clerks

Group 5: Service workers and shop and market sales workers

Personal and protective services workers
Models, salespersons and demonstrators

Group 6: Skilled agricultural and fishery workers

Market-oriented skilled agricultural and fishery workers
Subsistence agricultural and fishery workers

Group 7: Craft and related trade workers

Extraction and building trade workers
Metal, machinery and related trades workers
Precision, handicraft, printing and related trades workers
Other craft and related trades workers

Group 8: Plant and machine operators and assemblers

Stationary plant and related operators
Machine operators and assemblers
Drivers and mobile plant operators

Group 9: Elementary occupations

Sales and services elementary occupations
Agricultural, fishery and related labourers
Labourers in mining, construction, manufacturing and transport

Group 10: Armed forces

Armed forces

Group 11: Homemaker

Housewife/Househusband

Subject ID

☐☐ Centre # ☐☐☐ Community# ☐☐☐ Household # ☐☐ Subject #

Subject Initials ☐☐☐ F M L

31. In your opinion, do people generally help others not related to them in this community?
(check one only)

- ☐ This is common for people in my neighbourhood
- ☐ Infrequently, but it may happen
- ☐ Some adults would do this in our neighbourhood
- ☐ This would not occur in our neighbourhood

32. For day to day shopping for fruit and vegetables and other daily food items, where would you usually go?
(please list from closest location to farthest away)

a) Location: ______________________________ (include shop name and street)

b) ☐ In the community I live in ☐ Outside the community I live in (select one only, reseacher to allocate)

33. For other food and daily living items that you obtain on a weekly or less frequent basis, where would you usually go?*(please list from closest location to farthest away)*

a) Location: ______________________________ (include shop name and street)

b) ☐ In the community I live in ☐ Outside the community I live in (select one only, reseacher to allocate)

34. Approximately what percentage of the household's fruit and vegetables are grown by the household and hence, not bought from a shop?

☐☐☐ **%**

35. Details of participant

a) Age: ☐☐

b) Gender: ☐ Female ☐ Male

c) Smoking status: ☐ Current ☐ Former ☐ Never

d) Education: (check highest level completed only)**:**

- ☐ None
- ☐ Primary
- ☐ Junior high/secondary school
- ☐ Senior high/secondary schooll
- ☐ Trade school
- ☐ College/University

e) Durning your working life what was your main occupation**:** ______________________________

f) Please indicate which group best describes your main occupation (see facing page for codes)**:** ☐☐

g) What is/was the location of your workplace? ______________________________

h) ☐ In the community I live in ☐ Outside the community I live in (select one only, reseacher to allocate)

36. Interviewer name: ______________________________ **Date:** ☐☐☐☐ ☐☐ ☐☐
year month day
