# Supplementary material for: Environmental Profile of a Community's Health (EPOCH): An Instrument to Measure Environmental Determinants of Cardiovascular Health in Five Countries
Source: PLoS One. 2010 Dec 10;5(12):e14294. doi: 10.1371/journal.pone.0014294 (PMC3000812; doi:10.1371/journal.pone.0014294)
Supplement: Appendix S3 — EPOCH manual: version September 8, 2008 (0.28 MB PDF) [file pone.0014294.s003.pdf]

Manual for conducting community assessment with

EPOCH

Environmental Profile of a Community's Health

Contact information:
Dr. Clara K Chow
Population Health Research Institute
Hamilton General Hospital
237 Barton Street East
Hamilton ON L8L 2X2
Tel: 905 527 4322 extn 44206
Email: clara@phri.ca

Contents of the Manual

Introduction 3
Objectives 3
Relevant qualifications/ skills of the person conducting EPOCH 3
Preparation prior to community assessment 3
Structure of the EPOCH assessment 3
Defining the 'Community Area' to be assessed 3
Guidelines for defining the '*Community Area'* in the PURE study: 4
Planning the start point and '*Community Observation Walk'* of EPOCH I 4
Conducting EPOCH Part I – Direct Observations of the Community Environment 6
General information regarding documentation 6
Photographic assessment 6
Section 1 - Community demographics 6
Section 2 – Community Observation Walk 7
Observing the community for advertisements and availability of local shops: 7
Observations at store selling cigarettes 11
Observations at grocery store/ stores 12
Restaurant assessment 14
Photographic methods 15
Check list 16
Conducting EPOCH Part 2 – Survey of Community Awareness, Attitudes and Social Norms 17
General Instructions 17
Documentation and filling out the profile 17
Conducting the interview 17
Community tobacco environment 17
Community nutrition/ Physical activity environment 18
Community social environment 18
Additional questions 18

Introduction

Objectives

The aim of EPOCH is to provide a standardized and reliable instrument to profile a community for multiple social and environmental factors that can put that community at risk of higher levels of heart disease. The use of a standardized instrument will enable comparison of communities for a common list of environmental measures.

There are currently no tools that exist that measure the social and envrionmental factors of a community that relate to heart disease. In addition amongst instruments that measure some environmental determinants of heart disease, none have examined the applicability of environmental determinants to communities from different cultures and economics. The EPOCH instrument has been developed from a detailed review of the literature on social and environmental determinants, input from experts in the field and investigators from a range of countries.

Relevant qualifications/ skills of the person conducting EPOCH

This appraisal is to be conducted by a researcher/ research assistant who has a background in health-related research and has received training in EPOCH. This person should be familiar with the community to be assessed and able to speak the main language spoken in the community. It is ESSENTIAL that the researcher attends or views the training package just prior to the conducting an EPOCH assessment. For Part 1 - It is ESSENTIAL that the researcher spends time familiarising or re-familiarising themselves with all components of the EPOCH Part 1 tool immediately before attending

Preparation prior to community assessment

Structure of the EPOCH assessment

EPOCH is composed of two main questionnaires: **Part 1** – Direct Observations of the Community Environment and **Part 2** – Survey of Community Awareness, Attitudes and Social Norms. Part 1 – is composed of two main sections, the "Community demographics" section and the "Community Observation Walk".

Defining the 'Community Area' to be assessed

A common definition for "community" that is applicable globally is difficult to establish. The EPOCH tool has been designed for use in communities that have been defined as "a group of people who have common characteristics and reside in a specific area". Prior to conducting EPOCH assessment, it is important that the community's 'specific area' is defined.

Guidelines for defining the '*Community Area'* in the PURE study:

- For PURE - The *Community Area* to be defined for appraisal should encompass the residential addresses of participants recruited to the PURE study as well as their local services. Ideally participant's residential addresses and local services should be marked on a map and a rough circle with approximately a block (~100m) buffer drawn around them that encompasses the conglomerate of addresses/ services. The area within this circle is the area that is defined as the *'Community Area'* and is the area that should be referred to as the 'Community' in both EPOCH 1 and EPOCH 2. (There may be circumstances where this is difficult to do, and these should be discussed with the project office prior to commencement of the appraisal.). The map of the Community Area should be attached as instructed to the EPOCH 1 questionnaire.
- The first two questions in EPOCH Part I are designed to help define the Community Area. These two questions should be answered first and discussed with the project office prior to starting the appraisal of your community area.
- In communities that are "centered" it is likely that all information for the *'Community Observation Walk'* section will be collected in one central area. In communities that are not-centred, the *Walk* may be conducted in one area and the Shop assessments may be conducted in another area. The *'Community Observation Walk*' should usually be conducted in a selected area within the *Community Area*, where the majority of services in that community exist.
- The Community Area to be appraised should not overlap with other community areas being assessed.
- The borders of each Community Area should be clearly defined e.g. by the streets that border the area, or by natural borders such as a riverbank, or a cluster of postal code areas could be used.
- Ideally the population residing in the Community Area should have some similarities from local knowledge, in terms of sharing similar culture, being of similar socio-economics, accessing similar amenities/ goods and services.
- The size of Community Areas within a country is likely to be variable. However should not be too large (e.g. not more than 5 or so kilometres in diameter).
- Examples of Community Areas for RURAL communities: In rural areas of states of India or countries of South America the designated area may contain one large village; or a cluster of small villages may be combined if they share a similar area/ similar facilities/ similar infrastructure.
- Example of Community Areas for URBAN communities: Within one large city different community areas may not be separated by a great physical distance but be very different in other factors (e.g. socio-economics) which warrant them to be defined as two separate communities. In some large cities, there are 'slum areas' or 'high-income areas' grouping the wards or clustering postcodes within these areas is likely the best method of defining these community areas.

Planning the start point and '*Community Observation Walk'* of EPOCH I

The *'Community Observation Walk'* should be conducted in an area that people from that community would consider their central district for services and facilities in that Community Area. It is ESSENTIAL that the Community Observation Walk is pre-

planned. The start point should be decided in consultation with other researchers in your centre and the route of the walk planned.

The central start point: This should be in the busiest local thoroughfare of this area. It may or may not be the geographical centre of the community. A start point is selected to enable a standard method of data collection. Repeat assessments of this community area should start from the same point. A list of possible places that could be designated a 'start point' is listed in this question, select one or describe the point you have chosen in the space provided. Data needs to be collected at one of these points only.

The walking route: The route should be approximately 500 metres. Both sides of the street need to be observed; therefore a total walking distance of approximately 1 kilometre should be covered. A walk of approximately this length should be planned. In addition to a start point, you will need to identify a point where you turn around and assess the other side of the street. This point may be a cross-street or a specific address. If you have access to software such as Google maps, the length of the walk between two intersections or two addresses should be measured exactly. The walk may be along one length of street or may involve multiple turns and be along more than one street to stay within the busy area of the community. A pre-assessment of the community area through a casual walk or drive past the area you are planning to assess is highly encouraged to get this right. This should be drawn or printed and pasted in the appropriate space in the EPOCH 1 questionnaire.

Figure 1 – Planning a route

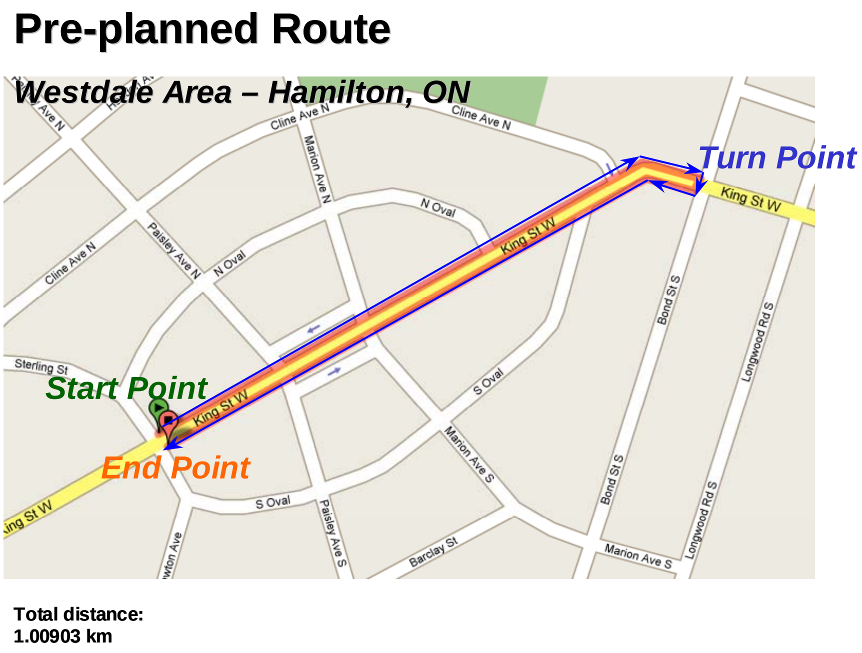

Conducting EPOCH Part I – Direct Observations of the Community Environment

General information regarding documentation

- The assessment should be filled out neatly in black pen or pencil. If it is difficult to keep the writing in the instrument neat as you are conducting this in the field, transcribe the answers to a new form when you return.
- No questions should be left blank/ unanswered
- Main question/ text/ statements in **bold.** Additional instructions in *italics*
- If questions are to be skipped, the instructions for these are in brackets in CAPITAL letters
- For certain countries, some additional questions have been added, for example in South Asia – questions are added regarding Beedi

Photographic assessment

There is a list of requested photos and these should be taken while conducting the EPOCH part 1 assessment. A series of photos will assist us in qualitatively comparing neighborhoods and will also assist communities in identifying the locations researchers have assessed. Photos need to be digital, carefully named, dated and stored. The list of photos is noted in the questionnaire in the Community Observation Walk section. Guidelines for photography are listed in a separate section. A letter of explanation is provided to give to shopkeepers. The following photographs are to be included in your assessment:

1. Photo of the street scene in each direction from the **start point** (a minimum of 4 photos)
2. Examples of **advertisements** that are classified in table 1 of Question 18 (please include one photo of each category of advertisement that is identified on your community observation walk)
3. Outside/ Front of shop of the **tobacco shop** visited
4. Outside/ Front of shop of the **grocery store** visited
5. Photo of **fruit and vegetable display** in the shop or in the stall (a minimum of 2 photos, 1 of fruits and one of vegetables)
6. Photograph the front of the **restaurant** visited

Section 1 - Community demographics

- The questions in the Community Demographics section are about the Community Area that has been defined. A researcher should complete this section. The researcher may obtain this information from a combination of sources including talking to a knowledgeable person living in the area, telephone books, internet resources or other local ressources – e.g. local tourist guides.
- The questions regarding facilities and transport services refer to the defined Community Area.
- Distances should be assessed as accurately as possible as distance to drive from the rural area to nearby towns etc. Again if Google maps or similar available, this is likely to be useful in calculating the distance between places.

- The average cost per unit area of residential property is best obtained from a local government source. Otherwise other potential sources are local real estate agents.

Sidewalk completeness and quality
In this section you are assessing the availability and quality of sidewalks on your walk. **Part a.** of the question questions asks you to rate the **completeness** of the sidewalk on your walk:

- Give a rating of **4** if sidewalk is complete on both sides of the street.
- Give a rating of **3** if sidewalk is complete on one side of street
- Give a rating of **2** if sidewalk is incomplete on both sides of street
- Give a rating of **1** if there are no sidewalks present. You would then skip part B of the question.

Part b. of the question asks you to rate the **quality** or maintenance of the sidewalks present:

- Give a rating of **4** if the sidewalks are very well maintained. This means the sidewalk has no bumps, cracks, holes and weeds
- Give a rating of **3** if the sidewalks are somewhat maintained. This means the sidewalk has few bumps, cracks, holes and weeds
- Give a rating of **2** if the sidewalks are undergoing repair
- Give a rating of **1** if sidewalks are poorly maintained. This means sidewalk has many bumps, cracks, holes and weeds

Section 2 – Community Observation Walk

- The walk should be pre-planned and mapped out (see section on Preparation prior to Community Assessment)
- The walk should be done in daylight/ regular working hours.
- The time and date of the assessment should be noted in the areas specified.
- The start point and address should be noted

Observing the community for advertisements and availability of local shops:

- The aim of this section is to count the total number of advertisements in different categories and shops/ local facilities in different categories.
- At the start of the walk, from a standing position in front of the designated start point, you are asked to systematically look in all directions around you and document the advertisements you see. Then do the same systematic observation of all directions from the opposite side of the street to see if you see anything you missed from the other side of the street. This should not be rushed.
- Take photos from the start point to capture the broad scene. These photos will be used to assess the quality of the neighbourhood.
- Also take close up photos of any specific examples of advertisements you see.
- From the start point proceed to walk ***slowly*** down the sidewalk of the street, noting down all that you see. It is best to stop and start as you walk down the road to observe the environment carefully. Depending on the density of the area you choose, the whole walk may take about 40 minutes.

Guidelines for counting advertisements:

Include all advertisements that you see. Include:

- Advertisements on poster boards, pasted on walls, pasted on bus stops, pasted on bus or cars that go by you as you do the walk
- Advertisements on shop windows and advertisements just inside windows within ~ 1 metre of the window that is clearly visible by people walking by (A4 paper size or greater)
- If you see advertisements pasted on boxes pressed against shop windows or on the pavement, include if advertisement is easily visible (e.g. A4 paper size).
- If there are multiple advertisements of the same type stuck one on top of each other or adjacent to each other on a single surface count as one advertisement.
- Advertisements may be on shopping trolleys on the pavement, and this can also be included if A4 size or greater, if there are a line of shopping carts/trolleys outside a store, do not count each shopping cart!
- If a poster board is two sided, with the same or different advertisement on each side, count this as two advertisements.
- Restaurants sometimes have their menu or menu items on the window. Do not include these as advertisements. For example, a Fast food chain may advertise their 'value meal deals' on the window – do not include these. However if Fast food chains advertise in nearby areas, i.e. on separated signs that are more than 5 metres from the restaurant shop front these can be included in the count.

Guidelines for counting shops

- Each store should be put into **one** category only. Do not count a shop more than once.
- If store seems to fit into more than one category, categorize it into the best option available.
- If convenience stores/ general stores sell cigarettes mark in: "Convenience store/general stores (with cigarettes)" only
- If convenience stores/ general stores do not sell cigarettes mark in: "Convenience stores/ general stores" only

Types of advertisements - Pictorial examples of the types of each advertisement are in the associated presentation. Brief notes on definitions are included here:

	Advertisements	Definition
A	Cigarette/ tobacco product advertisements	Advertisements that markets the sale of cigarette or tobacco products
B	Signs that prohibit smoking	One that prohibits smoking
C	Health promotion advertisements regarding the adverse effect of smoking/ tobacco cessation	Advertisements that promote the adverse health effects of smoking and/ or encourage people to not smoke e.g. smoking cessation advertisements
D	Health promotion advertisements regarding the adverse effect of alcoholic beverages	Advertisements that promote the adverse health effects of alcohol and/ or encourage people to drink e.g alcohol cessation advertisements
E	Snack food advertisements (e.g. chocolate, potato chips, fried sweet or salty snack foods – *researchers should discuss local examples with their teams*)	Advertisements that promote any type of snack foods e.g. chocolate, potato chips, locally made fried sweet or salty snack foods. This does not include fresh fruit or vegetable snacks advertisements. This does not include the advertisement of menu items on fast food stores.
F	Sugary drink advertisements (e.g. Coke, Pepsi, fruit juices, sports drinks, other well known brands)	Advertisements that promote non-alcoholic sweet drinks. Drinks that contain high content of sugar. Also known as soft drinks.
G	Non-commercial or Government health promotion advertisements promoting the health benefits of a good diet e.g. benefits of diet high in vegetables/ fruit[2]	Advertisements that promote the benefits of a good diet (e.g low in fat, high in vegetables/ fruit) or healthy foods (e.g. promote low-fat foods). In this section include only advertisements that have been sponsored by a governmental organization or a non-commercial organization.
H	Commercial Health promotion advertisements	As above, however sponsored by a commercial group.
I	Non-commercial HP (Physical Activity)	Advertisements that promote the benefits of physical activity/ sports/walking/exercise. In this section include only advertisements that have been sponsored by a governmental organization or a non-commercial organization.
J	Commercial Health Promotion (Physical Activity)	As above, however sponsored by a commercial group.
K	Alcoholic drink advertisements	Advertisements for alcohol containing drinks e.g. beer, wine, alcohol containing coolers/ sweet drinks.

Types of shops - Pictorial examples of the types of shops are in the presentation.

	Shops	Definitions
A	Vending machines (cigarettes)	This can include vending machines that you see inside shops, if you can see these from your walk.
B	Vendors/ street stands (cigarettes)	These are sellers of cigarettes that are non-permanent. They may be mobile, or they may have a semi-permanent stand that they can set up at different places or move to different locations. They don't usually pay for the rental of premises.
C	Convenience store/general stores (with cigarettes)	Convenience stores/ General stores sell a variety of items. They are not as large as a supermarket, often open long hours and sell a variety of foods and other goods. Mark stores that sell cigarettes along with other items.
D	Vending machines (snack foods)	Vending machines that sell snack foods, such as chips, chocolate, candies. This can include vending machines that you see inside shops, if you can see these from your walk.
E	Vending machines (sweet drinks)	Vending machines that sell sugary drinks such as cola, fruit juices, sport drinks etc. This can include vending machines that you see inside shops, if you can see these from your walk.
F	Vendors/ street stands/ shops that specialize in snack foods[3] (e.g. sweet stores, cake shops, ice-cream stands) [3]	Any type of store that their main business is the sale of snack foods. This may include sweet or salty snacks.
G	Convenience stores/ general stores (no cigarettes) [4]	These are similar to C but these include convenience stores or general stores that DO NOT sell cigarettes.
H	Supermarket	Large store that sells a combination of fresh and packaged goods. Stocks most kinds of groceries.
I	Fruit store/ street stand/ market store	This is a store/ street stand/ market store that specialises in sale of fruit and/or vegetables
J	Butcher/ meat store/ market store	Specialised in sale of raw meat
K	Bakery	Specialise in baked goods, such as bread, buns, etc.
L	Deli/ other specialty food store	Specialised in the sale of goods that typically have been treated such that they last longer e.g. smoked, salted meats, cheese, coffee beans, tea leaves, etc.
M	Alcohol speciality stores	Stores that sell only alcoholic products
N	Fast food restaurants[4]	Fast food restaurants are characterised by minimal service and by food that is supplied quickly after ordering. People queue to collect food. Food is NOT brought to the table. Typical examples include the international chains such as McDonalds or Kentucky Fry, but many local versions exist. It is best to make a list of the local examples and discuss these with the project office prior to commencements. (It is best that a photo of a locally relevant example is included in the training presentation)
O	Cafes/ fast casual restaurants	These are similar to fast-food in that it does not offer table service but promises somewhat higher quality of food and atmosphere. (http://en.wikipedi.org/wiki/Fast_casual_restaurant). You may order and pay at a counter. Often food is brought to your table. All these places have sit down areas.
P	Pubs / bars (that you can drink alcohol with or without a meal)	Main business is selling alcoholic beverages. They may or may not serve food. (It is best that a photo of a locally relevant example is included in the training presentation)
Q	Sit down restaurants	Sit down restaurant that offers full table service by wait staff who take your order at the table. (It is best that a photo of a locally relevant example is included in the training presentation)

Other things to be observed

	Other features of the community	Definitions
A	Public places Areas for recreation or physical activity- e.g. football pitches, parks, swimming pools	This includes any public space or area for recreation/ physical activity you can see from your walk route. e.g. park/ playground/ sport field/ public garden/ landscaped open space/ plaza square/ courtyard/ beach/ swimming pools
B	Street trees/ street flower beds	These are trees or flower beds that are located on the side of the pavement between the walker and the road, or may be at the centre of the road. Each tree is counted as one. Each flower bed is counted as one.

Observations at store selling cigarettes

- Observations only need to be made at one cigarette store. The outlet should be identified on your walk. If none available, go to the nearest place that sells cigarettes. If more than one available, visit the first store identified.
- Note the location of the shop carefully and photograph the front of the tobacco store
- Answer the questions regarding advertisements/ information available on tobacco. Refer to the following table for definitions.
- We ask you to document the cost of a standard brand "Marlboro". This is usually more expensive than most local brands. If a Marlboro is not available, other brands that may be included here include: Virginia Slims, Merit, Parliament, Benson &Hedges, L&M, Chesterfield, Lark, Cambridge, Basic. Other international brands not available in U.S. include: Apollo Soyuz, Bond Street, Caro, Diana, f6, Kazakstan, Klubowe, Longbeach, Multifilter, Muratti, Peter Jackson, Petra, Philip Morris, Polyot, SG, Vatra, Tobacco
- Counting brands of cigarettes. Include the broad types of brands here. If one brand has different types, e.g. different sizes or different strengths of cigarette, classify them all together as the same brand.

Definitions for in-store observation

	Term	Definitions
1	Point- of –sale advertising	Advertising at or near the store counter which is usually inside the store
2	Open display	Are the cigarettes displayed such that they are easy to see when entering the store, or are they under the counter/ in a drawer away from public display
3	Single unit cigarettes	Cigarettes sold separately. You can buy one cigarette at a time
4	Cheapest brand	Cheapest packet of 20 cigarettes that you can buy in this store
5	Health warning	Any warning on the cigarette packet that warns against the potential harm of cigarette on your health
6	Snack foods	This includes any type of snack foods – food for light meals or eating in between meals – e.g. chocolate, potato chips, donuts, and cakes, locally made fried sweets or salty snack foods. This does not include fresh fruit or vegetable snacks.
7	Sugary drinks	These included sweet drinks e.g. Coke, Pepsi, fruit juices, sport drinks etc.

Observations at grocery store/ stores

In this section, the objectives are to

1. Cost the items on the grocery list
2. Make general observations regarding the food-purchasing environment
3. Assess the availability of a list of common fruit and vegetables
4. Assess the attractiveness/ quality of fruit and vegetables though a photograph of a the fruit/ vegetable display

- Provide the best description of the food-purchasing environment in the defined Community Area. Refer to presentation slides for examples
- Identify a nearby local supermarket or group of stores where people in this community would buy fruits and vegetables or other daily food items. This may be one store or multiple stores. Mark the type of store or stores you need to visit to buy the items listed in the shopping list in Q28.
- Visit the store to obtain food prices and document the location of the store. If more than one store write the location of the nearest store and describe the advertisements you see here.
- Photograph the front of the store

Guidelines for costing of foods

- Follow the guidelines in the questionnaire to identify a supermarket or group of shops that sell all the grocery items that are on the list. If more than one exist, go to the place that is most commonly frequented by locals in the area. If all items are not in one supermarket or one store walk to nearby stores/ vendors within a 1000 metre radius of the store you have identified. If you are unable to find the items, write not available
- Collect the costs of each item. If there is more than one kind of an item, choose the kind that is the cheapest, non-sale item and collect the price of this.
- The weights of items may be different from what is stated. If this is the case, collect the weight of the item such that a cost for 1kg of the item can be calculated.
- A medium sized egg is approximately 55 to 60 grams. Many countries sell eggs in a box of a twelve eggs, hence the cost of 1 egg will be the total cost divided by 12. 1 kilogram of eggs is equal to about 17 or 18 medium sized eggs. The format of this item can be modified by country to make this data easier to collect.

Guidelines for assessing availability

For fruits and vegetables, there are separate tables containing a list of each. The list provided is a combination of fruits and vegetables obtained commonly across a number of PURE countries. Not all fruit/ vegetables are likely to be available in your country or available in the season you are conducting your assessment. There may also be other fruit available, or similar fruits. If the fruits/ vegetables are similar mark the boxes that best fit these fruits/ vegetables. Do not remove items from the list. Mark all the fruit or vegetables available on this list.

Guidelines for assessing quality

- To assess quality there are a set of items regarding the presentation of fruit and vegetables in the store.
- Fruit and vegetables are visible from the outside of the store if you can see them through the window while standing outside.
- To assess if fruit and vegetables have been damaged, check for obvious bruising, items that are rotten, inedible or of poor quality. If three or more are damaged note 'yes'. If less than this mark 'no'.
- In some stores, fruit or vegetables is displayed in boxes where there is separate packing for each fruit to prevent bruising of the fruit. Fruit or vegetables may also be separately wrapped to prevent damage. If there are 3 or more kinds of fruit in this store where this has been done, mark yes here.
- The final item to assess quality will be evaluated from photos taken of the fruit display. Please photograph the fruit and vegetable displays in the shop as per photographic guidelines. See appendix.

Other notes/ definitions relevant to this section:
The local food-purchasing environment - Photographic examples are included in the accompanying slide presentation.

Packaged snack food item:
Choose an item from the grocery store you visit that is packaged snack food, such as chips, crackers, chocolate etc. Look to see if there are any nutritional labels on the can and answer the questions regarding the labels contents. In addition copy any additional labels or cut the label and include it with your EPOCH assessment.

Restaurant assessment

- Identify a typical low-cost restaurant. Ideally this should be identified on your Community Observation Walk. However if there is no restaurant on your walk, then go to the nearest restaurant.
- A restaurant is a venue in which food is eaten outside the home. A place that only specialises in take-away food is not included in this definition.
- Classify this restaurant according to the definitions given. Some restaurants may seem to fit into more than one category, if so, classify according to its main business.
- Photograph the front of the restaurant

	Term	Definitions
A	Sit down restaurant	A restaurant that offers full table service by wait staff who take your order at the table (e.g. *…please insert some locally relevant examples for your location*)
B	Bars/ Pubs	A restaurant that sells a full range of alcoholic beverages
C	Fast casual restaurant	A restaurant similar to fast-food in that it does not offer table service, but promises somewhat higher quality of food and atmosphere. You may order and pay at a counter. Often food is brought to your table, but not always. (http://en.wikipedi.org/wiki/Fast_casual_restaurant). E.g. Noodle shops,
D	Fast food restaurants & Take-away or delivery places	A restaurant characterized by minimal service and by food that is supplied quickly after ordering. Food is commonly cooked in bulk in advance and kept hot, or reheated to order. Fast food is often finger food that can be eaten quickly without cutlery e.g. McDonalds, Kentucy Fried Chicken … *give other local examples for your location*
D	Outdoor food stall with seating	An outdoor food restaurant that sells pre-cooked or food cooked to order. These have minimal or no facilities.
F	Other	Please use this if the restaurant identified does not fit into the above categories and give details to describe this restaurant.

Assess for indicators of health promotion

- Healthy menu options – These are options that are highlighted for being good for your health. E.g – low fat menu items
- Increased portion size – In some restaurants there are offers to increase or 'up-size' your food/ drink order for a small price. These are often marketed as 'value for money' options. Terms that many be used include: "all-you-can eat/ drink for one price", 'super-size', 'jumbo', 'grande', 'supreme', 'king size' … *give local examples for your location when training.*

Menu (Optional)

If possible obtain a copy of the menu in the restaurant. This could be a photocopy, a digital copy or a carefully transcribed copy.

Photographic methods

It is very important that the photos taken are photographed in a standard manner.
Please use a digital camera for all photography. The digital camera should be at least 2 megapixel. For 2 megapixel camera use the maximum quality settings. You will need at least a 1 gigabyte memory card in your camera to complete all the photography per community.
Additional guidelines are included below. Please also refer to the training presentation for photo examples.

- **Lighting -** Community assessments should be conducted through daylight working hour, hence all outdoor photographs should be taken in day-light. You can photography on sunny or overcast days, but try and avoid photographing while raining. After rain light can be good for photography.
- **Photos of scenes** - e.g. street scene in each direction from start point. Do not zoom in, take photos in normal or wide angle. When taking photos in multiple directions around the starting point, ensure photos overlap. If you have a 'stitch assist' feature on the camera, it will be helpful to use to stitch together overlapping photos. Photos of street scenes should include the road or street in the photo. If there are tall buildings above street level that you would like to capture, a second set of photos may be required.
- **Photographs of shop fronts** – Try and stand back to capture the whole shop front. Try to avoid using flash, as this will shine of the windows. Photograph from a 30 to 40 degree angle to more easily capture the whole shop front. The shop front should take up at least 50% of your photos, do not include too much of the surrounding scenery in these photos. See examples.
- **Photographing indoors** – for fruit and vegetable displays, these may be indoors. You may require flash here. Try not to photograph through glass. Photograph from a 30 to 40 degree angle to more easily capture the whole display. The fruit and vegetable display should take up at least 70% of your photograph. You might need multiple photos to capture the nature of the fruit and vegetable display in a shop.
- **Photos focussing on specific objects/ items** – For photos of street signs or advertising, it is important to keep the photo in careful focus to enable reading of the sign/ advertising. You are likely to require zoom for these photos, try to avoid using flash as this will likely reflect from the signs and make them difficult to read. The sign advertising should take up at least 50% of the photograph, and preferably more.

Check list

- ❑ Read the manual with assessment tool
- ❑ Review the direct assessment tool in detail
- ❑ Review the training presentation in detail
- ❑ Defined the community area and discussed this with project investigators
- ❑ Assigned a central starting point
- ❑ Planned the walking route

Once completed, you may start your direct community assessment. Good Luck!

Conducting EPOCH Part 2 – Survey of Community Awareness, Attitudes and Social Norms

General Instructions

- Part 2 - is to be conducted by interview
- The sampling framework for the individuals to be interviewed will depend on local resources. However to gain a broadly representative view of community members, it is recommended that the sample includes men and women, younger and older persons and smokers and non-smokers.

Documentation and filling out the profile

- The main question/ text/ statements in **bold**. Additional instructions in *italics* are not to be read out.
- Questions are generally of two formats:
    - Select best answer – tick one box with the item that best fits with the respondent's answer to the question.
    - Yes/ No, or Yes/ No/ Unsure – for each numbered statement, answer yes or no. In some cases there is also the option of unsure.
- All questions are to be read out unless there is a specified skip. If questions are to be skipped, the instructions for these are in brackets in CAPITAL letters

Conducting the interview

- Start by introducing your self with a statement like this – ***"Thank you for agreeing to participate in this survey. This survey aims to collect information about your community as well as attitudes towards health. When I refer to the community I mean …(describe the community defined in part I and show the participant the map). "***
- The community area that you describe is identical to that you have defined in your preparation for the community assessment (see section on preparation). You should hence have a map of the community to show to the person you are interviewing, or if it is a phone interview, you should be able to clearly describe the community area – in terms of its geographical boundaries.

Community tobacco environment

- Question 1 to 4, and Q7,8, 9 of this section aims to evaluate what the community participant has observed in their community with regards to behaviour of smokers. These questions are not aimed at evaluating the awareness of the participants of the laws, but what they have observed in their own communities.
- Question 5 aims to evaluate if there are any rules about smoking in the home. Rules could be set by you or your family members with respect to smoking. Choose the best statement that describes the situation in your home. If no formal rules have been set, but there are implicit rules, answer the question with respect to these implicit rules.
- Question 6, 10 - 12 aims to evaluate the attitudes of community participants towards smoking. They seek a participant's opinion of smoking in public places or by

different groups of people in the community. The participant is asked to report on what they think is the general consensus towards these attitudes.

- Question 14 to 18 aims to evaluate awareness of laws and support programs for tobacco cessation that influence the participant's community.
    - Youth are those < 18 years of age
- Question 19 aims to evaluate the knowledge of participants of the health effects of smoking.
- Question 20 and 21 are specifically targeted at smokers. Question 21 is to estimate how much people pay for cigarettes. If people do not buy loose cigarettes (that is they only buy cigarettes in packets) this does not have to be filled out, and this is applicable visa versa.

Community nutrition/ Physical activity environment

- Question 22 to 25 aims to evaluate what the community participant has observed in their community with regards to food and drink advertising and labelling of foods
- Question 26 aims to evaluate the knowledge of the participant of the health effects of diet and physical activity behaviours.
- Question 27 to 29 aim to evaluate the awareness of the participant of laws that influence the sale and labelling of food/ beverages

Community social environment

- Question 30 and 31 aim to evaluate some social norms in the community

Additional questions

In these questions 32 and 33 we aim to find out where people shop.

- Please document the location and address of the shop/ shops that people usually go to shop for fruits and vegetables or other daily food items. The aim of documenting this location is such that we can mark this on a map if required in the future and calculate the exact distance from the persons residence to the shop they usually attend. We can then find out how far people are prepared to go for their daily shopping items.
- For part b of this question, according to the definition of the Community Area you have set, decide whether this location is within the Community Area or outside the Community Area (refer to definition of Community Area).
- Question 33 is similar to the above. If people go shopping for specialty or other items regularly on a weekly, fortnightly or monthly basis, then document the location they go. If they just go to the same location as in Question 32, document the same location.
- Question 34 in this section is asking how much of the household's fruit and vegetables are grown and hence not bought. In most urban areas, fruit and vegetables are likely to be all bought; the answer to this question would be 0%. However in some rural areas a proportion of fruit and vegetables may be grown on their property and hence not directly paid for.
- The final question aims to collect basic descriptive data on the participant, much of this is repeated from the baseline questionnaire, however we do also require location of work place. Again we are looking for an address here, such that in the future we can mark this on a map and calculate the distance a person travels to and from work.
