# Supplementary material for: Environmental Profile of a Community's Health (EPOCH): An Instrument to Measure Environmental Determinants of Cardiovascular Health in Five Countries
Source: PLoS One. 2010 Dec 10;5(12):e14294. doi: 10.1371/journal.pone.0014294 (PMC3000812; doi:10.1371/journal.pone.0014294)
Supplement: Appendix S4 — Reliability testing by country (0.25 MB DOC) [file pone.0014294.s004.doc]

**Appendix T**able 1 Reliability testing in communities from Canada

|  | **Environmental attribute** | **ICC** | **95% CI** | **No. of communities** |
| --- | --- | --- | --- | --- |
| ***Community demographics*** | |  |  |  |
| 1 | Cost of residential land | 0.59 | (0.11 , 0.92) | 6 |
| 2 | Number of public transportation services | 0.90 | (0.84 , 0.94) | 39 |
| 3 | Maximum daily frequency of public transportation | 0.98 | (0.96 , 0.99) | 39 |
| 4 | Number of types of public services/education facilities | 0.87 | (0.80 , 0.93) | 39 |
| 5 | Number of types of community infrastructure | 1.00* |  | 39 |
| 6 | Number of types of community health facilities | 0.89 | (0.82 , 0.94) | 39 |
| 7 | Sidewalk completeness and quality scale | 0.93 | (0.88 , 0.96) | 39 |
|  |  |  |  |  |
| ***Community observation walk*** | |  |  |  |
| 1 | Number of tobacco advertisements | 0.80 | (0.69 , 0.88) | 39 |
| 2 | Number of signs prohibiting smoking | 0.95 | (0.91 , 0.97) | 39 |
| 3 | Number of health promotion advertisements | 0.81 | (0.71 , 0.89) | 39 |
| 4 | Number of snack food advertisements | 0.92 | (0.87 , 0.96) | 39 |
| 5 | Number of sugary drink advertisements | 0.95 | (0.92 , 0.97) | 39 |
| 6 | Number of alcoholic drink advertisements | 0.93 | (0.88 , 0.96) | 39 |
| 7 | Number of places to buy cigarettes | 0.93 | (0.88 , 0.96) | 39 |
| 8 | Number of places to buy snack foods | 0.92 | (0.87 , 0.95) | 39 |
| 9 | Number of stores selling food | 0.95 | (0.92 , 0.97) | 39 |
| 10 | Number of places to buy alcohol | 0.89 | (0.81 , 0.93) | 39 |
| 11 | Number of restaurants | 0.98 | (0.97 , 0.99) | 39 |
| 12 | Number of parks and street trees | 0.73 | (0.59 , 0.83) | 39 |
|  |  |  |  |  |
| ***Tobacco store assessment*** | |  |  |  |
| 1 | In-store tobacco advertisements | 0.64 | (0.48 , 0.78) | 39 |
| 2 | In-store smoking cessation promotion | 0.56 | (0.37 , 0.72) | 39 |
| 3 | Number of tobacco brands | 0.38 | (0.19 , 0.58) | 39 |
| 4 | Number of sizes of cigarette pack available | 0.35 | (0.15 , 0.56) | 39 |
| 5 | Price of cheapest pack of cigarettes | 0.53 | (0.35 , 0.70) | 39 |
| 6 | Price of Marlboro/international brand | 0.50 | (0.30 , 0.68) | 34 |
| 7 | Number of health warnings on cigarette packs | 0.39 | (0.19 , 0.58) | 39 |
|  |  |  |  |  |
| ***Grocery store assessment*** | |  |  |  |
| 1 | Point of sale unhealthy food advertising | 0.61 | (0.44 , 0.76) | 39 |
| 2 | Point of sale healthy food advertising | 0.74 | (0.61 , 0.85) | 39 |
| 3 | Fruit and vegetable display quality scale | 0.98 | (0.96 , 0.99) | 39 |
| 4 | Number of types of fruits available | 0.87 | (0.79 , 0.92) | 39 |
| 5 | Number of types of vegetables available | 0.89 | (0.83 , 0.94) | 39 |
| 6 | Price of fruit | 0.84 | (0.75 , 0.91) | 39 |
| 7 | Price of vegetables | 0.78 | (0.67 , 0.87) | 39 |
| 8 | Price of other products | 0.83 | (0.73 , 0.90) | 39 |
| ***Restaurant assessment*** | |  |  |  |
| 1 | Healthy menu options (yes/no) | 0.28 | (0.09 , 0.50) | 39 |
| 2 | Main salad or vegetable dishes (yes/no) | 0.31 | (0.11 , 0.52) | 39 |
| 3 | Buffet service (yes/no) | 0.34 | (0.14 , 0.54) | 39 |
| 4 | Option to increase portion size (yes/no) | 0.32 | (0.13 , 0.52) | 39 |

**Appendix T**able 2 Reliability testing in communities from China

|  | **Environmental attribute** | **ICC** | **95% CI** | **No. of communities** |
| --- | --- | --- | --- | --- |
| ***Community demographics*** | |  |  |  |
| 1 | Cost of residential land | 1.00* |  | 18 |
| 2 | Number of public transportation services | 0.85 | (0.72 , 0.94) | 19 |
| 3 | Maximum daily frequency of public transportation | 0.26 | (-0.02 , 0.57) | 18 |
| 4 | Number of types of public services/education facilities | 0.62 | (0.37 , 0.82) | 19 |
| 5 | Number of types of community infrastructure | 0.61 | (0.36 , 0.81) | 19 |
| 6 | Number of types of community health facilities | 0.80 | (0.63 , 0.91) | 19 |
| 7 | Sidewalk completeness and quality scale | 0.75 | (0.55 , 0.88) | 19 |
|  |  |  |  |  |
| ***Community observation walk*** | |  |  |  |
| 1 | Number of tobacco advertisements | 0.57 | (0.30 , 0.79) | 19 |
| 2 | Number of signs prohibiting smoking | 0.77 | (0.59 , 0.90) | 19 |
| 3 | Number of health promotion advertisements | 0.42 | (0.13 , 0.69) | 19 |
| 4 | Number of snack food advertisements | 0.51 | (0.24 , 0.75) | 19 |
| 5 | Number of sugary drink advertisements | 0.77 | (0.59 , 0.90) | 19 |
| 6 | Number of alcoholic drink advertisements | 0.56 | (0.29 , 0.78) | 19 |
| 7 | Number of places to buy cigarettes | 0.67 | (0.43 , 0.84) | 19 |
| 8 | Number of places to buy snack foods | 0.83 | (0.67 , 0.92) | 19 |
| 9 | Number of stores selling food | 0.85 | (0.72 , 0.93) | 19 |
| 10 | Number of places to buy alcohol | 0.43 | (0.15 , 0.69) | 19 |
| 11 | Number of restaurants | 0.92 | (0.84 , 0.97) | 19 |
| 12 | Number of parks and street trees | 0.76 | (0.57 , 0.89) | 19 |
|  |  |  |  |  |
| ***Tobacco store assessment*** | |  |  |  |
| 1 | In-store tobacco advertisements | 0.41 | (0.14 , 0.68) | 19 |
| 2 | In-store smoking cessation promotion | 0.68 | (0.45 , 0.85) | 19 |
| 3 | Number of tobacco brands | 0.71 | (0.50 , 0.86) | 19 |
| 4 | Number of sizes of cigarette packs available | 0.28 | (0.01 , 0.59) | 19 |
| 5 | Price of cheapest pack of cigarettes | 0.10 | (-0.15 , 0.43) | 19 |
| 6 | Price of Marlboro/international brand | 0.91 | (0.78 , 0.97) | 11 |
| 7 | Number of health warnings on cigarette packs | 1.00* . |  | 19 |
|  |  |  |  |  |
| ***Grocery store assessment*** | |  |  |  |
| 1 | Point of sale unhealthy food advertising | 0.78 | (0.60 , 0.90) | 19 |
| 2 | Point of sale healthy food advertising | 0.63 | (0.37 , 0.82) | 19 |
| 3 | Fruit and vegetable display quality scale | 0.51 | (0.23 , 0.75) | 19 |
| 4 | Number of types of fruits available | 0.79 | (0.62 , 0.91) | 19 |
| 5 | Number of types of vegetable available | 0.70 | (0.47 , 0.86) | 19 |
| 6 | Price of fruit | 0.82 | (0.66 , 0.92) | 18 |
| 7 | Price of vegetables | 0.68 | (0.46 , 0.85) | 19 |
| 8 | Price of other products | 0.63 | (0.38 , 0.82) | 19 |
| ***Restaurant assessment*** | |  |  |  |
| 1 | Healthy menu options (yes/no) | 0.70 | (0.48 , 0.86) | 19 |
| 2 | Main salad or vegetable dishes (yes/no) | 0.71 | (0.49 , 0.87) | 19 |
| 3 | Buffet service (yes/no) | 1.00* |  | 19 |
| 4 | Option to increase portion size (yes/no) | 0.67 | (0.43 , 0.84) | 19 |

*No observer variation

**Appendix T**able 3 Reliability testing in communities from India

|  | **Environmental attribute** | **ICC** | **95% CI** | **No. of communities** | |
| --- | --- | --- | --- | --- | --- |
| ***Community demographics*** | |  |  |  |  |
| 1 | Cost of residential land | 1.00* |  | 15 | |
| 2 | Number of public transportation services | 0.87 | (0.73 , 0.95) | 15 | |
| 3 | Maximum daily frequency of public transportation | 0.98 | (0.96 , 0.99) | 15 | |
| 4 | Number of types of public services/education facilities | 0.89 | (0.76 , 0.96) | 15 | |
| 5 | Number of types of community infrastructure | 0.95 | (0.89 , 0.98) | 15 | |
| 6 | Number of types of community health facilities | 0.94 | (0.86 , 0.98) | 15 | |
| 7 | Sidewalk completeness and quality scale | 0.97 | (0.93 , 0.99) | 15 | |
|  |  |  |  |  | |
| ***Community observation walk*** | |  |  |  |  |
| 1 | Number of tobacco advertisements | 0.94 | (0.87 , 0.98) | 15 | |
| 2 | Number of signs prohibiting smoking | 0.00 | (-0.22 , 0.35) | 15 | |
| 3 | Number of health promotion advertisements | 1.00*. |  | 15 | |
| 4 | Number of snack food advertisements | 0.89 | (0.77 , 0.96) | 15 | |
| 5 | Number of sugary drink advertisements | 0.96 | (0.92 , 0.99) | 15 | |
| 6 | Number of alcoholic drink advertisements | 1.00* |  | 15 | |
| 7 | Number of places to buy cigarettes | 0.91 | (0.81 , 0.97) | 15 | |
| 8 | Number of places to buy snack foods | 0.91 | (0.81 , 0.97) | 15 | |
| 9 | Number of stores selling food | 0.96 | (0.90 , 0.98) | 15 | |
| 10 | Number of places to buy alcohol | 0.96 | (0.91 , 0.99) | 15 | |
| 11 | Number of restaurants | 0.82 | (0.63 , 0.93) | 15 | |
| 12 | Number of parks and street trees | 1.00* |  | 15 | |
|  |  |  |  |  | |
| ***Tobacco store assessment*** | |  |  |  |  |
| 1 | In-store tobacco advertisements | 0.95 | (0.89 , 0.98) | 15 | |
| 2 | In-store smoking cessation promotion | 0.00 | (-0.22 , 0.35) | 15 | |
| 3 | Number of tobacco brands | 0.99 | (0.98 , 1.00) | 15 | |
| 4 | Number of sizes of cigarette packs available | 0.92 | (0.83 , 0.97) | 15 | |
| 5 | Price of cheapest pack of cigarettes | 0.87 | (0.73 , 0.95) | 15 | |
| 6 | Price of Marlboro/international brand | 0.45 | (-0.09 , 0.91) | 5 | |
| 7 | Number of health warnings on cigarette packs | 1.00* | ( . , .) | 15 | |
|  |  |  |  |  | |
| ***Grocery store assessment*** | |  |  |  |  |
| 1 | Point of sale unhealthy food advertising | 0.96 | (0.92 , 0.99) | 15 | |
| 2 | Point of sale healthy food advertising | 1.00* |  | 15 | |
| 3 | Fruit and vegetable display quality scale | 0.98 | (0.95 , 0.99) | 15 | |
| 4 | Number of types of fruits available | 0.99 | (0.98 , 1.00) | 15 | |
| 5 | Number of types of vegetable available | 0.99 | (0.97 , 1.00) | 15 | |
| 6 | Price of fruit | 0.99 | (0.98 , 1.00) | 12 | |
| 7 | Price of vegetables | 1.00 | (0.99 , 1.00) | 8 | |
| 8 | Price of other products | 1.00 | (1.00 , 1.00) | 15 | |
| ***Restaurant assessment*** | |  |  |  |  |
| 1 | Healthy menu options (yes/no) | 1.00* |  | 10 | |
| 2 | Main salad or vegetable dishes (yes/no) | 1.00* |  | 10 | |
| 3 | Buffet service (yes/no) | 1.00* |  | 10 | |
| 4 | Option to increase portion size (yes/no) | 1.00* |  | 10 | |

*No observer variation

**Appendix T**able 4 Reliability testing in communities from Colombia and Brazil

| **Environmental attribute** | **ICC** | **95% CI** | **No. of communities** |
| --- | --- | --- | --- |
| ***Community demographics*** |  |  |  |
| 1 Cost of residential land | 0.83 | (0.68 , 0.92) | 20 |
| 2 Number of public transportation services | 0.92 | (0.84 , 0.96) | 20 |
| 3 Max. daily frequency of public transportation | 0.95 | (0.90 , 0.98) | 20 |
| 4 Number of types of public services/education facilities | 0.85 | (0.71 , 0.93) | 20 |
| 5 Number of types of community infrastructure | 0.82 | (0.67 , 0.92) | 20 |
| 6 Number of types of community health facilities | 0.80 | (0.63 , 0.91) | 20 |
| 7 Sidewalk completeness and quality scale | 0.93 | (0.85 , 0.97) | 20 |
|  |  |  |  |
| ***Community observation walk*** |  |  |  |
| 1 Number of tobacco advertisements | 0.66 | (0.44 , 0.83) | 20 |
| 2 Number of signs prohibiting smoking | 0.33 | (0.06 , 0.61) | 20 |
| 3 Number of health promotion advertisements | 0.36 | (0.09 , 0.63) | 20 |
| 4 Number of snack food advertisements | 0.83 | (0.66 , 0.93) | 20 |
| 5 Number of sugary drink advertisements | 0.62 | (0.38 , 0.81) | 20 |
| 6 Number of alcoholic drink advertisements | 0.21 | (-0.03 , 0.50) | 20 |
| 7 Number of places to buy cigarettes | 0.69 | (0.47 , 0.85) | 20 |
| 8 Number of places to buy snack foods | 0.67 | (0.44 , 0.84) | 20 |
| 9 Number of stores selling food | 0.55 | (0.30 , 0.77) | 20 |
| 10 Number of places to buy alcohol | 0.53 | (0.26 , 0.75) | 20 |
| 11 Number of restaurants | 0.63 | (0.40 , 0.82) | 20 |
| 12 Number of parks and street trees | 0.89 | (0.77 , 0.95) | 20 |
|  |  |  |  |
| ***Tobacco store assessment*** |  |  |  |
| 1 In-store tobacco advertisements | 0.57 | (0.31 , 0.78) | 20 |
| 2 In-store smoking cessation promotion | 0.86 | (0.73 , 0.94) | 20 |
| 3 Number of tobacco brands | 0.81 | (0.65 , 0.91) | 20 |
| 4 Number of sizes of cigarette packs available | 0.78 | (0.59 , 0.90) | 20 |
| 5 Price of cheapest pack of cigarettes | 0.92 | (0.84 , 0.96) | 20 |
| 6 Price of Marlboro/international brand | 0.92 | (0.85 , 0.97) | 18 |
| 7 Number of health warnings on cigarette packs | 0.38 | (0.10 , 0.66) | 20 |
|  |  |  |  |
| ***Grocery store assessment*** |  |  |  |
| 1 Point of sale unhealthy food advertising | 0.38 | (0.11 , 0.66) | 20 |
| 2 Point of sale healthy food advertising | 0.17 | (-0.09 , 0.49) | 20 |
| 3 Fruit and vegetable display quality scale | 0.49 | (0.23 , 0.73) | 20 |
| 4 Number of types of fruit available | 0.51 | (0.22 , 0.75) | 20 |
| 5 Number of types of vegetable available | 0.54 | (0.26 , 0.76) | 20 |
| 6 Price of fruit | 0.50 | (0.11 , 0.66) | 20 |
| 7 Price of vegetables | 0.72 | (0.51 , 0.87) | 20 |
| 8 Price of other products | 0.81 | (0.64 , 0.91) | 20 |
| ***Restaurant assessment*** |  |  |  |
| 1 Healthy menu options (yes/no) | 0.21 | (-0.06 , 0.52) | 20 |
| 2 Main salad or vegetable dishes (yes/no) | 0.22 | (-0.05 , 0.53) | 20 |
| 3 Buffet service (yes/no) | 0.85 | (0.71 , 0.93) | 20 |
| 4 Option to increase portion size (yes/no) | 0.54 | (0.28 , 0.76) | 20 |

*No observer variation
